# Systematic analysis of photo/sko-regulated germination and post-germination development of shallow photodormant seeds in *Nicotiana tabacum* L.

**DOI:** 10.3389/fpls.2022.1042981

**Published:** 2023-01-04

**Authors:** Qiyuan Liu, Zhenhua Li, Min Zhang, Shuai Dong, Pingping Yang, Jie Zhang, Eddison Loades

**Affiliations:** ^1^ College of Agriculture, University of Guizhou, Guiyang, Guizhou, China; ^2^ China National Tobacco Corporation (CNTC) Key Laboratory of Molecular Genetics, Guizhou Academy of Tobacco Science, Guiyang, Guizhou, China; ^3^ Department of Biological Sciences, Royal Holloway, University of London, London, United Kingdom

**Keywords:** seed germination, imbibition, radicle protrusion, cotyledon expansion, transcriptome, proteome

## Abstract

**Introduction:**

Light is a major environmental factor in regulating germination and post-germination development of shallow photo-dormant seeds in *Nicotiana tabacum* L. (tobacco). However, its molecular mechanism remains largely unclear.

**Methods and results:**

In this study, we compared the phenotypes of the seeds germinated under light and dark, and systematically investigated their regulatory networks by integrating transcriptomic and proteomic data. Under light, the germination increased ~25%, the length of the hypocotyl shortened ~3 cm, and the apical hook disappeared. 9, 161, 342 differentially expressed genes (DEGs) and 128, 185, 81 differentially expressed proteins (DEPs) were regulated by light in the development stage of seed imbibition, radicle protrusion and cotyledon expansion respectively. 0, 19 and 1 co-up-regulated and 1, 30 and 64 co-down-regulated DEGs (DEP) were observed in the three stages, respectively. Of them, 2S albumin large chain, was down-regulated by light in imbibed seed. Oleosin 18.5 kDa (OLEO1) and Glyceraldehyde-3-phosphate dehydrogenase (GAPA1), Oxygen-evolving enhancer protein 1-1 and anchloroplastic (PSBO1), hub genes (proteins) in protein-protein interaction network (PPI), were downregulated and up-regulated in germinated seeds by light, respectively. OLEO1, a hub gene (proteins), was down-regulated by light in post-germination seedling.

**Conclusion:**

These results systematically revealed the molecular networks regulated by light during germination and post-germination development of shallow photo-dormant tobacco seeds.

## Introduction

Light is one of the major environmental factors in regulating seed germination. Seeds could be divided into three categories according their response to white light. Photophilic seeds, germinate after a short time exposure to light, while photophobic seeds, only germinate under dark. Non-photoblastic seeds, germinate well in both light and darkness. Hormones ABSCISIC ACID (ABA) and GIBBERELLINS (GA) are thought to be involved in the regulation of germination in all three categories seed (Sawada et al., 2008; Guo et al., 2018; Mérai et al., 2019). In *Arabidopsis*, the interaction between light, ABA and GA has been elaborately illuminated during seed germination, the biosynthesis of GA is stimulated by light, and in turn high levels of GA suppresses the synthesis of ABA (Vanstraelen & Benková, 2012; de Wit et al., 2016). PHYTOCHROME B (PHYB) and (or) PHYA is activated by red (R) and (or) far red (FR) light and mediates the degradation of PHYTOCHROME INTERACTING FACTOR1 (PIF1), and that relieves the suppression of PIF1 to GA anabolism and signal, which results in GA level increasing and suppresses ABA anabolism (Oh et al., 2006; Oh et al., 2007), promoting germination. In the dark, PHYB and (or) PHYA is inactivated and allows PIF1 accumulation, leading to suppression of GA signaling and stimulation of ABA anabolism (Kim et al., 2008; Oh et al., 2009), which results in ABA levels increasing and GA levels decreasing, inhibiting germination.

Following germination, seeds alternatively develop into etiolated or green seedling when they were exposed to dark or light. Light inhibits hypocotyls elongation and promotes apical hook disappearance and cotyledon unfolding. *pif* mutants exhibit a constitutive photomorphogenic phenotype even in darkness, as characterized by a shorter hypocotyl, open cotyledons, and apical hook defects (Shin et al., 2009; Leivar and Quail, 2011). In the dark, PHYs are inactivated that leads to the accumulation of PIFs (Gu et al., 2017; Qiu et al., 2019) and CONSTITUTIVE PHOTOMORPHOGENIC1/SUPPRESSOR OF PHYA-105 complexes (COP1/SPAs) (Huang, 2014; Hoecker, 2017), which promotes skomorphogenesis. Upon light, PHYs are activated to regulate the degradation of PIFs and destabilization of COP1/SPA complexes (Balcerowicz et al., 2011; Pacín et al., 2013; Sheerin et al., 2015), resulting in the accumulation of HY5, which promotes photomorphogenesis (Osterlund et al., 2000). In addition, *pif* mutants exhibit a apical hook defects phenotype even in darkness (Shin et al., 2009; Leivar and Quail, 2011). PIFs transcription factors promote hook curvature by activating HLS1 transcription (Zhang et al., 2018).

Seed germination begins imbibition and completes at the protruding radicle, and subsequently enters post-germination stage. Their molecular mechanisms of each development stage have been extensively studied in *Arabidopsis*, which recognized as a model plant to study biology mechanism of plant growth. However, the molecular mechanism of germination is quite different among species, especially between photophilic, non-photoblastic and photophobic seeds. In tobacco, photodormant seeds cannot germinate in the dark, which was taken as an model plant for studying the mechanism of seed photodormancy (Leubner-Metzger, 2002; Kucera et al., 2005). In our prevent study, we noticed fresh seeds of minority varieties in tobacco (shallow-photodormant type, dark-germination is more than 50%) that matured in shading environments could germinate independent of light (Dong et al., 2022). In this study, seeds of shallow-photodormant type were selected to study the molecular mechanisms of photo/sko-regulated germination and post-germination development by integrating transcriptomic and proteomic data. In particular, the PPI network was used to reveal the role of light at seed imbibition, radicle protrusion and cotyledon expansion stages.

## Materials and methods

### Materials

Seeds of tobacco variety Y85 were provided by the Tobacco Research Institute of Guizhou. All the seeds were harvested 40 days after pollination, and mechanically dried at 40 ℃ for 36 hours in a hot air dryer (Jiang su, China). After threshing, the seeds were stored at room temperature, and all the experiments were conducted within 1 month. Seeds were incubated in two environments (light expose and continue dark) and developed in three phases (DAI2, 4, 6) (**Table 1**), containing three replicates of 18 samples were used for transcriptome and proteome sequencing, respectively.

**Table 1:** The light treatment of germinated seed and developed seedling in tobacco.

Light treatment	Days after imbibition (DAI)			Compare (COM)	
	2	4	6	(DAI 4/2)	(DAI 6/4)
Continue dark	DY85-2	DY85-4	DY85-6	COM4 (DY85-4 VS DY85-2)	COM 6 (DY85-6 VS DY85-4)
12h light/dark cycle	LY85-2	LY85-4	LY85-6	COM5 (LY85-4 VS LY85-2)	COM 7 (LY85-6 VS LY85-4)
Compare (Light/dark)	COM1 (LY85-2 VS DY85-2)	COM2 (LY85-4 VS DY85-4)	COM3 (LY85-6 VS DY85-6)		

The first letter L or D in each set represents seeds of Y85 that germinated under light or dark, and numbers 2 and 4 after the “-” indicate the days after seed imbibition.

### Seed treatment and germination test

The seeds were first disinfected by soaking in 0.5% copper sulfate solution for 15 min. Subsequently, the seeds were rinsed three times with distilled water. Finally, the disinfected seeds were dried with absorbent paper. 0.8% agar solutions were evenly poured into petri dishes to make agar germination beds. 100 seeds were evenly placed on the surface of the agar bed using a 10*10 grid, with three replicates for each treatment. The petri dishes were subsequently placed in an artificial climate chamber (Ningbo, China) and cultivated at 25°C, 80% humidity, 12 h photoperiod or constant darkness.

After 9 days' cultivation in greenhouse, the effects of different light environment treatments on tobacco seed germination related traits (germination rate, germination potential) were investigated. The formula of germination rate is:

Germination potential = The number of germinated seeds within 7 days / The number of tested seeds × 100%;

Germination rate = The number of seeds germinated within 14 days / The number of tested seeds× 100%.

### Phenotypic date acquisition

Seed or seedling phenotype was identified as described by Li et al., (2019). In brief, three replicates of 100 seeds per treat were positioned in an imaging chamber, and the images were obtained daily through the Scanalyzer HTS (German, LemnaTec Company). Images were processed using Lemna Grid, which is an image analysis component of the LemnaTec HTS system. The total perimeter and area of whole seeds (seedlings) were measured by using the images from DAI1 to DAI 9. The perimeter and (or) area of radicle, hypocotyl and cotyledons were measured by using the images from DAI6 to DAI 9 images. In addition, the caliper length of the cotyledons (reflect the curvature of hooks) was also measured during this time.

### Transcriptome sequencing

Transcriptome sequencing and data analysis refer to our previously published method (Dong et al., 2022). Briefly, total RNA of each sample (see materials section) was extracted through RNAprep pure Plant Kit (Tiangen, Beijing, China). The quality of the RNA was determined by using a Nanophotometer Spectrophotometer (IMPLEN, California, USA) and an Agilent Bioanalyzer 2100 system (Agilent Technologies, CA, USA). The libraries were constructed through a TruSeq Stranded mRNA LT Sample Prep Kit (Illumina, CA, USA). The transcriptome sequencing was performed by OE Biotech Co., Ltd. (Shanghai, China). The libraries were sequenced by using an Illumina HiSeq X Ten platform, and 150 bp paired-end reads were generated. Raw reads were processed through Trimomatic (Bolger et al., 2014). Clean reads were mapped to the tobacco genome using HISAT2 (Kim et al., 2015). Fragments per kilobase million (Roberts et al., 2011) value for each gene was calculated using Cufflinks (Trapnell et al., 2010), and their read counts were obtained using HTSeq-Count (Anders et al., 2015). DEGs analysis was performed using the DESeq (2012) R package (Love et al., 2018). p-values < 0.05, fold change (FC) < 0.5 or > 2 and FPKM≥2 were used as thresholds for significantly DEGs. A principal component analysis (PCA) was performed on the expression profiles using the R(PCA) function.

### Proteome sequencing

Proteome sequencing and data analysis refers to Wang (2016). In Brief, ~1g sample (see materials section) was weighed, powdered in liquid nitrogen and transferred into 700 μL of extraction buffer. Then, 7×protease inhibitor solution (Roche Applied Science, Indianapolis, IN) were added, mixed completely, and shaken for 30 min at 4°C. Subsequently, the mixture was centrifuged at 12,000g for 10 min at 4°C, and supernatant was transferred to a new tube and the protein concentration was determined by the Brad-Centration method. Trypsin digestion and iTRAQ analysis were performed by OE Biotech Co., Ltd. (Shanghai, China). For the identification of DEPs and FC were calculated automatically by Mascot. For statistical analysis, a two-tailed t-test was used and a p-value was calculated for each protein, which was conducted by Mascot ,too. At last, only proteins with FC > 2 or < 0.5 and p-values < 0.05 were considered significantly different. A principal component analysis (PCA) was performed on the expression profiles using the R(PCA) function.

### Combined analysis of transcriptome and proteome

In this study, the protein and mRNA were from the same reference genome (Kim et al., 2015), and the combined analysis was performed between the protein and its corresponding mRNA according to the gene ID. Cor-Up-regulated and Cor-Down-regulated represent DEGs (DEPs) that are simultaneously up-regulated and down-regulated, respectively. Gene Ontology (GO) enrichment of Cor-Up-regulated and Cor-Down-regulated DEGs (DEPs) were analyzed through the R package based on hypergeometric distribution. GeneMANIA online software (https://genemania.org/) was used to construct PPI networks and the networks can reveal the relationships between genes, including co-expression, physical interactions, co-localization and inter-gene interactions. Cor-Up-regulated and Cor-Down-regulated DEGs (DEPs) of each COM were used to construct 6 PPI networks.

### Quantitative RT-PCR

Total RNA of seeds and seedlings was extracted using a TIANGEN RNA prep pure plant plus kit (Tiangen, Beijing, China). The purity and concentration of the RNA was assessed using a NanoDrop-2000 spectrophotometer (Thermo Science, MA, USA) and its integrity was assessed using an Agilent 2100 Bioanalyzer (Agilent Technologies, CA, USA). Purified RNA (1 µg per sample) was reverse transcribed to first-strand cDNA using a cDNA Reverse Transcription Kit (PrimeScript^TM^ RT Master Mix, Takara). RT-PCR was performed using TaKaRa TB Green® *Premix Ex Taq*™ II (Tli RnaseH Plus). Fluorescence Quantification Kit 20 µl reaction system as follows: 10 µL TB *Green Premix Ex Taq* (2×) (Tli RnaseH Plus), 0.4µL ROX reference dye (50×), 2µl of cDNA diluted to 40 ng/µL, 0.8 µl of upstream and downstream primers (**Table S1**), respectively, and made up to 20µl using double distilled water. RT-PCR reaction conditions: pre-denaturation for 0.5 min at 95°C, followed by at 95℃ for 5 s, 58℃ for 30 s and cycling 40 times. Relative expression levels of each gene were determined using a Step One Plus real-time PCR instrument with three replicates per treatment. Relative expression levels were calculated according to the published method (Livak and Schmittgen, 2001).

## Results and analysis

### Phenotypic differences of seed geminated under light and dark in tobacco

**Figure 1:**
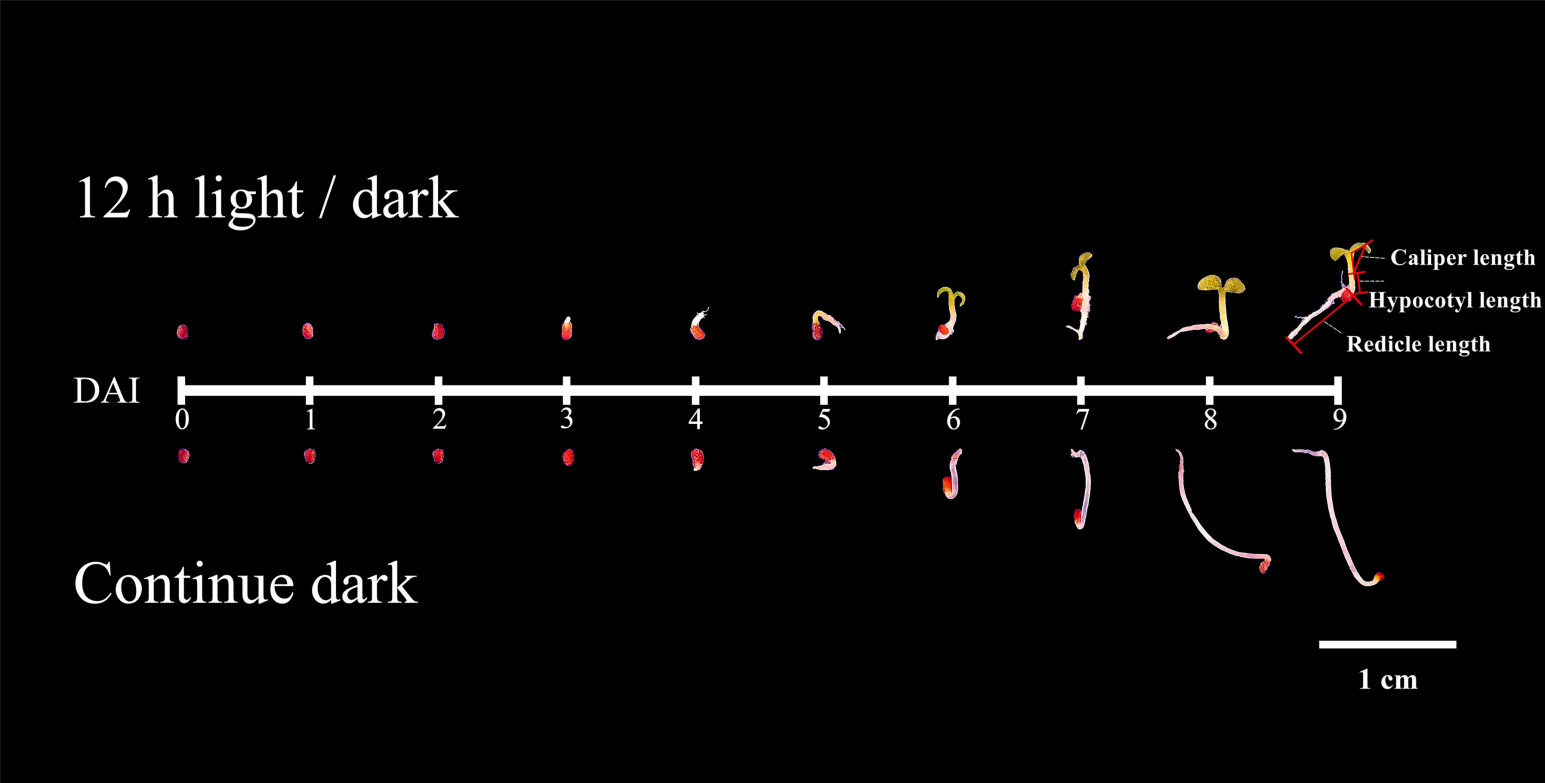
Phenotypes of seed germinated under light and dark.

In this study, we compared the developmental dynamics of tobacco seeds germinated under 12h light/dark cycles and continuous dark. According to phenotype, the development process could be divided into two stages (**Figure 1**), the germination stage (DAI1- 1 days after imbibition to DAI4- 4 days after imbibition /DAI5- 5 days after imbibition), and the post-germination development stage (DAI6- 6 days after imbibition to DAI9- 9 days after imbibition). Seed germination finished on DAI4 in light, while it taken 5 days in dark (**Figure 1**).

**Figure 2:**
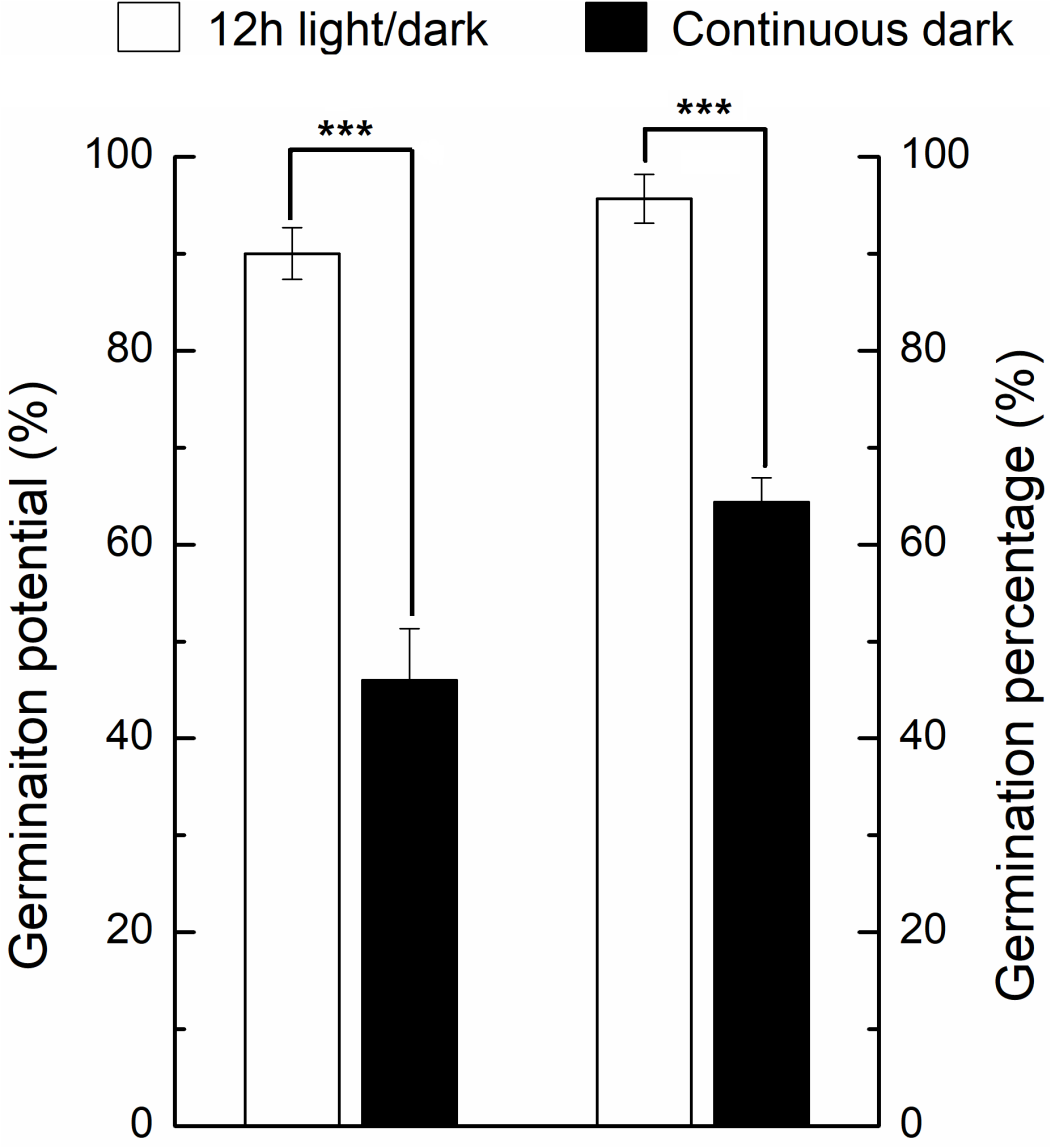
Germination percentage and germination potential of seeds under light and dark conditions. * indicates the significance of the test. * means significant at level 0.05; ** means significant at 0.01 level; *** means significant at 0.001 level. Same as below.

Under light, the germination percentage was ~90%, which was significantly higher than the dark germination of ~65% (**Figure 2**). Before DAI2, there were no significant differences of phenotype between light and dark treatments (**Figure 1**). From DAI6, the areas and diameters of the seedlings grown in continuous dark were significantly higher to that of 12h light/dark treatment, but the difference gradually decreased from DAI8 onwards (**Figures S1**, **S2**).

**Figure 3:**
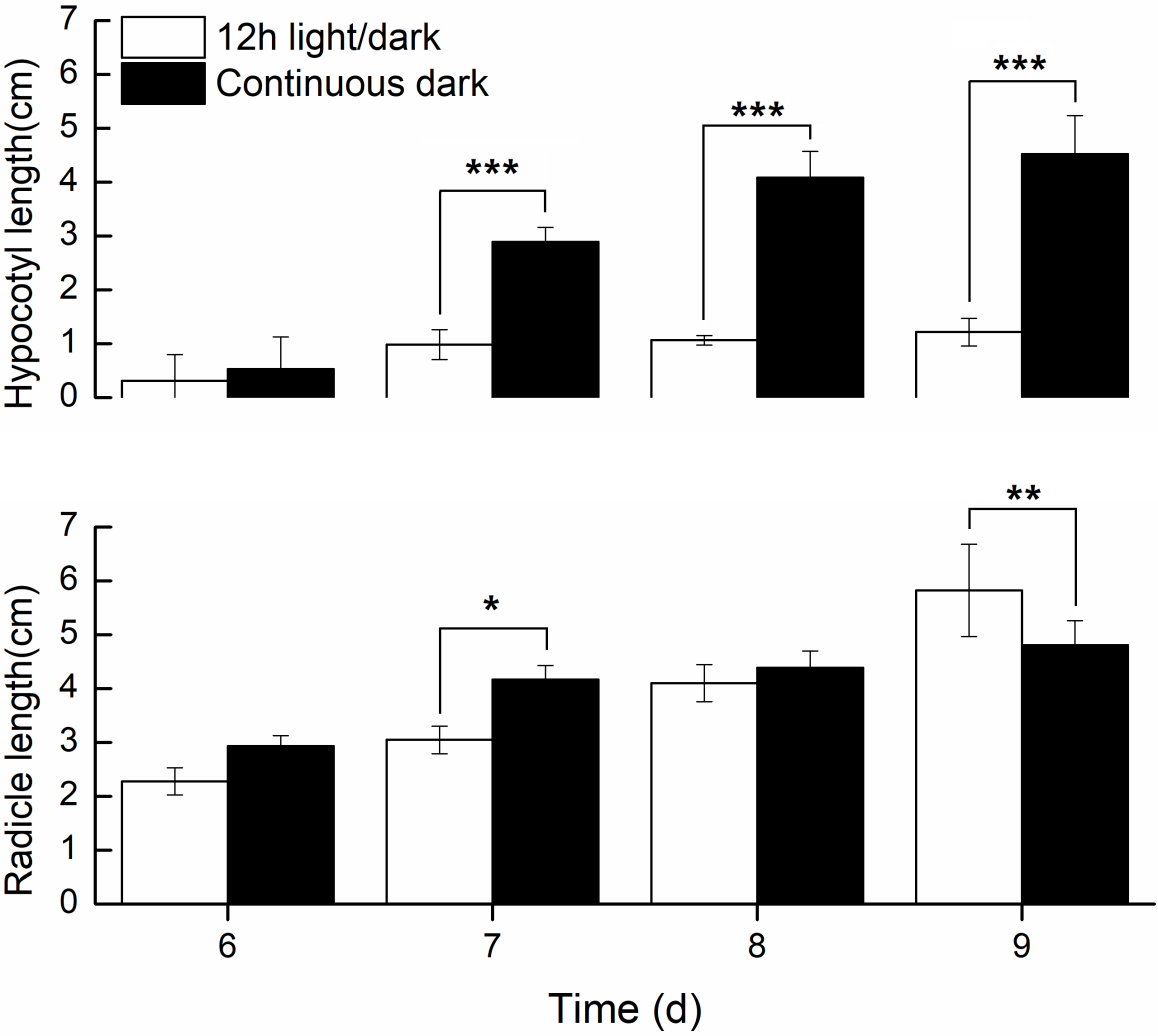
Radicle and hypocotyls lengths developed in light and dark.

**Figure 4:**
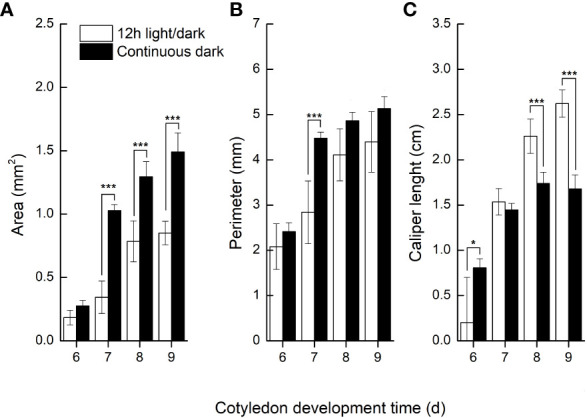
Cotyledon traits developed in light and dark.

We further compared the phenotypic differences of radicles, hypocotyls and cotyledons developed under light and dark from DAI6 (radicle protrusion) to DAI9 (cotyledon expansion). Hypocotyls lengths were several times longer (**Figure 3**), and the cotyledon areas were significantly larger under dark (**Figures 4A, B**). However, cotyledon curvatures were larger under light (**Figure 4C**).

### Transcriptome and proteome differences of seeds germinated under light and dark in tobacco

**Figure 5:**
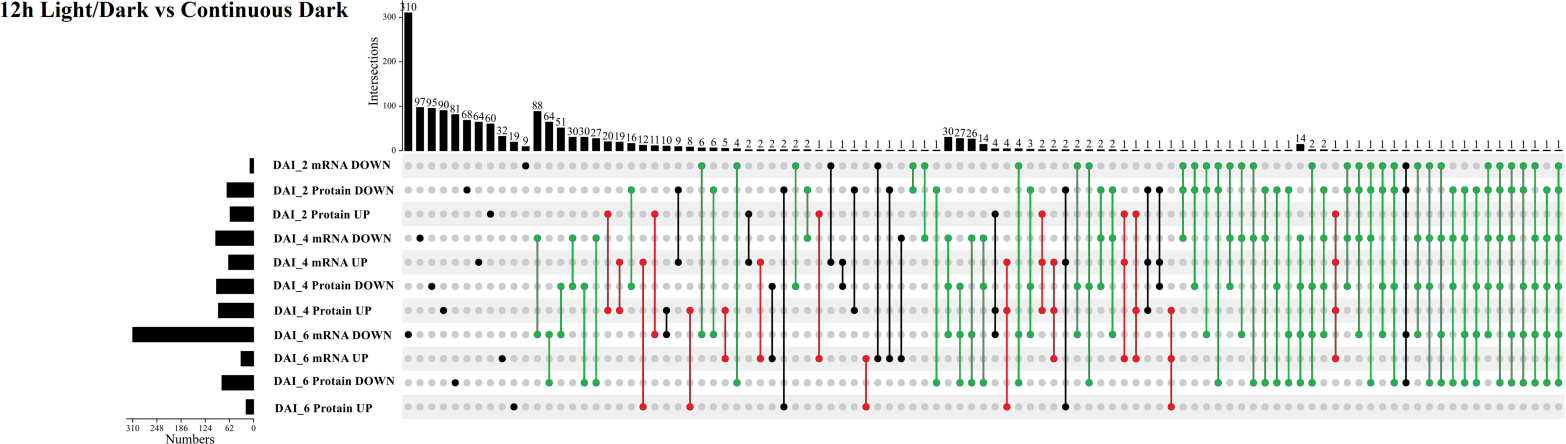
Multiple comparisons of light-regulated DEGs (DEPs) in seed germination and post-germination developmental stages. The histogram on the left represents the number of co-up and co-down regulated DEGs (DEPs) within each comparison. The histogram on the top represents the number of co-up or -down regulated DEGs (DEPs) from multiple comparisons. Multiple comparisons were displayed by lines and linked dots.

**Figure 6:**
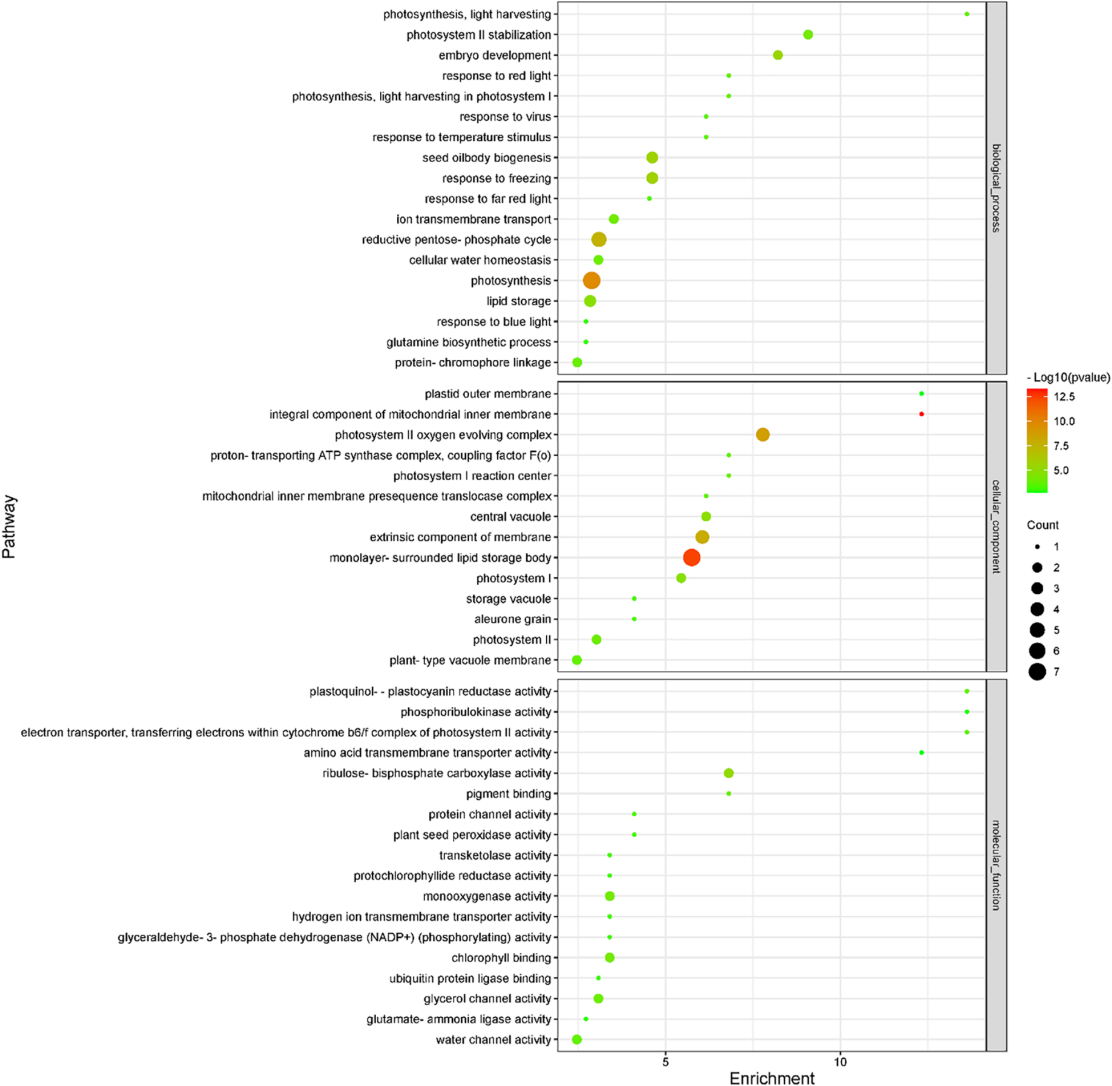
Enriched GO terms of light-regulated pathways at radicle protrusion stage. The size of the circle represented the number of genes enriched in the pathway, and the color of the cluster represents the confidence interval, and *p*-value was calculated from hypergeometric test.

Subsequently, we compared transcriptomic and proteomic data of seeds germinated under light and dark, a total of 7 combinations (COMs) were accomplished (**Table 1**). In COM1, 9 DEGs were identified, 0 and 9 of which were up- and down-regulated (**Figure S3A**). 128 DEPs were identified, 60 and 68 of which were up- and down-regulated (**Figure S3B**). 0 and 1 DEGs (DEPs) was co-up- and co-down-regulated (**Figure 5**), and 0 pathways were enriched. In COM2, there were 161 DEGs, 64 and 97 of which were up- and down-regulated (**Figure S3A**). 185 DEPs were identified, 90 and 95 of which were up- and down-regulated (**Figure S3B**). 19 and 30 DEGs (DEPs) were co-up- and co-down-regulated (**Figure 5**), and 21 pathways were enriched (**Figure 6**). In COM3, 342 DEGs were identified, 32 and 310 of which were up- and down-regulated (**Figure S3A**). 81 DEPs were identified, 19 and 64 of which were up- and down-regulated (**Figure S3B**). 1 and 30 DEGs (DEPs) were co-up- and co-down-regulated, and 16 pathways were enriched (**Figure S4**).

We also compared transcriptomic and proteomic data in three develop stage. 445 (50) up-regulated and 224 (49) down-regulated genes (proteins) were detected in COM4 (**Figure S5A**). Of them, 15 co-up-regulated and 24 co-down-regulated regulated genes (proteins) were identified (**Figure S5A**), and 9 pathways were enriched (**Figure S6**). 556 (50) up-regulated and 394 (49) down-regulated genes (proteins) were detected in COM5 (**Figure S5B**). Of them, 12 co-upregulated and 27 co-down-regulated genes (proteins) were identified (**Figure S5B**), and 6 pathways were enriched (**Figure S7**). 214 (246) up-regulated and 702 (180) down-regulated genes (proteins) were detected in COM6 (**Figure S5A**). Of them, 92 co-up-regulated and 37 co-down-regulated genes (proteins) were identified (**Figure S5A**), and 50 pathways were enriched (**Figure S8**). 203 (184) up-regulated and 949 (231) down-regulated genes (proteins) were detected in COM7 (**Figure S5B**). Of them, 63 co-up-regulated and 111 co-down-regulated genes (proteins) were identified (**Figure S5B**), and 57 pathways were enriched (**Figure S9**).

**Figure 7:**
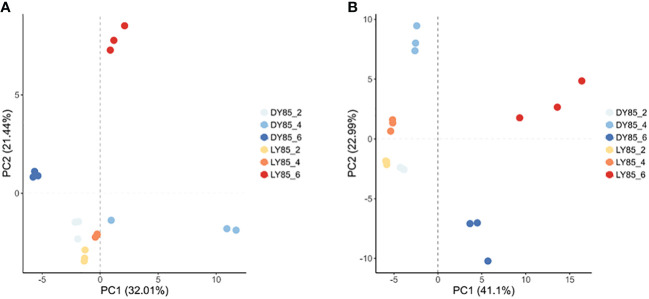
PCA plots of light-regulated germination and post-germination development. They were plotted with DEG **(A)** and DEP **(B)**, respectively. The consistency and diversity of the samples were observed through PCA. Each point in the figure represents a repetition, and different colors represent the difference of light treatment or development stage.

Principal component analysis (PCA) was used to analyze the diversity and similarity of samples from both light treatment and developmental perspectives. According to light treatment, the samples of LY85_2 and DY85_2 were gathered together by DEGs and DEPs, while LY85_6 and DY85_6 were completely separated by DEGs and DEPs (**Figures 7A, B**). LY85_4 and DY85_4 were gathered together by DEGs (**Figure 7A**), but not by DEPs (**Figure 7B**). According to develop stage, the samples of DY85_2, DY85_4 and DY85_6 were completely separated by DEGs and DEPs (**Figures 7A, B**), while the samples of LY85_2 and LY85_4 were completely separated by DEPs (**Figure 7B**), but not by DEGs (**Figure 7A**). Samples of LY85_4 and LY85_6 were completely separated by DEGs and DEPs (**Figures 7A, B**).

### Systematic analysis of germination and post-germination development when seeds incubated under light and dark in tobacco

**Figure 8:**
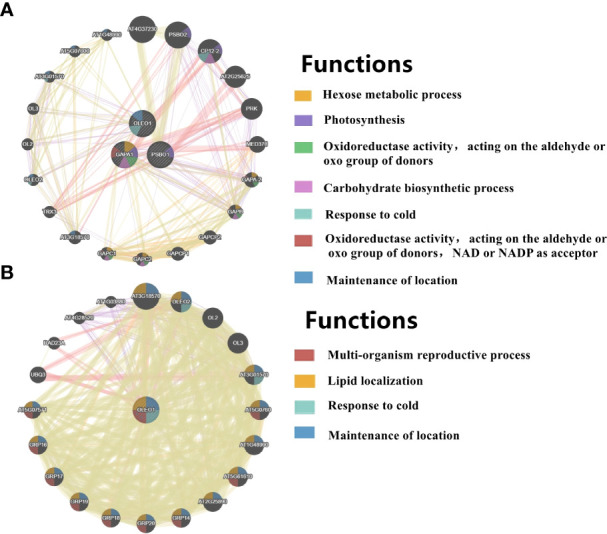
PPI networks of light-regulated germination and post-germination development, **(A)** radicle protrusion and **(B)** cotyledon expansion stage. The protein-protein interaction network for DEGs (DEPs) was analyzed using the GeneMANIA database during seed germination and post-germination development. At least 20 most frequently changed neighboring genes were shown. Each network node represented a gene. The node color represented the possible functions of these respective genes.

**Figure 9:**
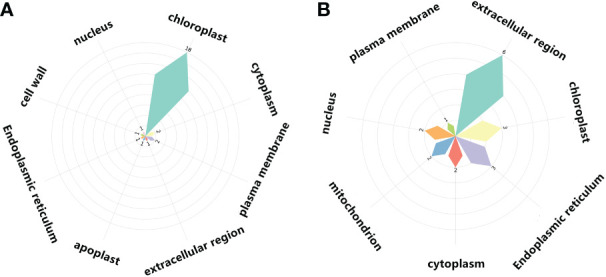
Cellular components of light-regulated germination and post-germination development, **(A)** Radicle protrusion and **(B)** cotyledon expansion stage. Petal size showed the number of cellular components enriched, and the number of signaling pathways enriched in cellular components was also listed.

The expression patterns of co-up- and down-regulated DEGs (DEPs) were analyzed by using UpSetR, and a total of 64 patterns were identified among COM1, COM2 and COM3 (**Figure 5**). 47 patterns were identified between COM4 and COM6 (**Figure S5A**) and 53 patterns between COM5 and COM7 (**Figure S5B**). GeneMANIA software was used to analyze associations in terms of co-expression, physical interactions and predicted pathway, and 6 PPI networks were constructed (**Figure 8** and **Figure S10**). GO analysis was used to identify of the cellular components of each COM (**Table 1**), and 6 component diagrams were constructed (**Figure 9** and **Figure S11**).

### Regulatory networks as seeds imbibed in light and dark

In COM1, we only identified one co-expressed genes (proteins) of the 2S albumin large chain, which was down-regulated by light in imbibed seed. 2S albumin is clearly as a seed storage protein and abundantly expressed in endosperm, whose biological functions include food reserve for germination and as an inhibitor of α-amylase to inhibit pre-germination during seed development. Because insufficient number of co-expressed genes (proteins) was identified, PPI network and cellular components diagrams cannot be established in COM1.

### Regulatory networks as radicles protruded in light and dark

In COM2, GAPA1, PSBO1, OLEO1 were the hubs of these 23 genes (proteins) (**Figure 8A**). Of them, GAPA1 and PSBO1 were up-regulated, while OLEO1 was down-regulated by light (**Table 2**). Their biological functions mainly included regulation of hexose metabolism process, photosynthesis, oxidoreductase activity, and so on (**Figure 8A**). For instance, GAPA1, GAPA2, GAPAB, GAPC1 and GAPC2 were consider to be involved in regulating of hexose metabolic process, and they participated in regulating photosynthesis together with PSBO2 and CP12-2. Notably, 18 of these 23 genes (proteins) were localized in the chloroplast (**Figure 9A**).

**Table 2:** Expression patterns of hub genes (proteins) during germination and post-germination development in light and dark.

Gene ID	Annotation	Compare (COM)							Biological functions
		1	2	3	4	5	6	7	
Nitab4.5_0000600g0090.1	OLEO1	None	Down	Down	UP	Up	Down	Down	Seed germination; Seed oil body biogenesis
Nitab4.5_0004979g0020.1	GAPA1	None	UP	None	None	UP	UP	UP	Glucose metabolism process; Reduced pentose phosphate cycle
Nitab4.5_0000090g0100.1	PSBO1	None	UP	None	None	UP	UP	UP	Photosynthesis, light reaction; Photosystem ll assembly; Photosystem ll stabilization
Nitab4.5_0000023g0130.1	MAN1	None	None	Down	UP	UP	Down	Down	Organic substance metabolic process
Nitab4.5_0002214g0100.1	GAD	None	None	None	UP	UP	UP	UP	Glutamate catabolic process
Nitab4.5_0000181g0120.1	PIP2-4	None	None	None	UP	UP	UP	None	Hydrogen peroxide transmembrane transport; Root hair elongation
Nitab4.5_0001485g0170.1	ATPC	None	None	None	None	UP	Down	UP	Proton motive force-driven ATPsynthesis
Nitab4.5_0000337g0200.1	FTSH1	None	None	None	UP	UP	UP	None	PSll associated light-harvesting complex ll catabolic process
Nitab4.5_0000059g0010.1	GLN2	None	None	None	UP	UP	UP	UP	Glutamine biosynthetic process
Nitab4.5_0000527g0230.1	RCA	None	Up	None	UP	UP	UP	UP	Response to light stimulus

COM1 (LY85-2 VS DY85-2), COM2 (LY85-4 VS DY85-4), COM3 (LY85-6 VS DY85-6), COM4 (DY85-4 VS DY85-2), COM5 (LY85-4 VS LY85-2), COM 6 (DY85-6 VS DY85-4), COM 7 (LY85-6 VS LY85-4).

In COM4 and COM5, the identical PPI network was constructed. Mannan endo-1,4-beta-mannosidase 1 (MAN1) was the hub of these 21 genes (proteins) respectively (**Figures S10A, C**), which was up-regulated at radicle protrusion stage in both light and dark (**Table 2**). MAN1, together with MAN3, MAN4, MAN5, MAN6 and MAN7, were predicted to regulate mannosidase activity and hydrolase activity of hydrolyzing O-glycosyl compounds and glycosyl bonds (**Figures S10A, B**). 6, 6, 5, 5 and 4 genes (proteins) were localized in cytoplasm, extracellular region, nucleus, chloroplast and endoplasmic reticulum, while 7, 7, 5, 5 and 4 genes (proteins) were localized in cytoplasm, nucleus, chloroplast, endoplasmic reticulum, extracellular region (**Figures S11A, B**).

### Regulatory networks as cotyledon expanded in light and dark

In COM3, OLEO1 was the hub of these 21 genes (proteins), which was down-regulated by light (**Table 2**). OLEO1, together with other genes were predicted to regulate maintenance of location, response to cold, multi-organism reproductive process, lipid localization (**Figure 8B**). 6, 3 and 3 genes (proteins) were localized in extracellular region, chloroplast and endoplasmic reticulum (**Figure 9B**).

In COM6, PSBO1, GAPB, CPBP1, RCA, GLN2, GAPA1, FTSH1, ATPC, RRF, PIP2-4, MAN1, RPL2, GAD1, and PSBS were the hubs of these 34 genes (proteins) (**Figure S10C**), and their expression patterns were shown in **Table 2**. The functions of 34 genes (proteins) are mainly involved in regulating glucose metabolic process, photosynthesis, generation of precursor metabolites and energy, and so on (**Figure S10C**). 66, 13, and 11 genes (proteins) were localized in chloroplast, ribosome, cytoplasm (**Figure S11C**).

In COM7, PSBO1, GAPB, CPBP1, PSBQ1, RCA, GLN2, ATPC, GAPA1, MAN1, OLEO1, MAN5, RPL2 and PSBS were the hubs of these 33 genes (proteins) (**Figure S10D**), and their expression patterns were shown in **Table 2**. The functions of 33 (gene) proteins are mainly involved in regulating photosynthesis, generation of precursor metabolites and energy, light harvesting (photosynthesis), and so on (**Figure S10D**). 49, 15 and 14 genes (proteins) were localized in chloroplast, extracellular region, cytoplasm (**Figure S11D**).

Overall, the PPI networks differed more in developmental stages than in light treatment during seed germination and post-germination development. 7 of the 10 hub genes (proteins) were up-regulated from seed imbibition to radicle protrusion under either light or dark conditions. 7 of the 10 hub genes (proteins) were up-or down-regulated from radicle protrusion to cotyledon expansion under either light or dark conditions. However, 0, 4 and 2 hub genes (proteins) up-or down-regulated by light at seed imbibition, radicle protrusion and cotyledon expansion, respectively. The effect of light on seed imbibition was significantly less than on radicle protrusion and cotyledon unfolding from the PPI networks.

### RT-PCR validation

**Figure 10:**
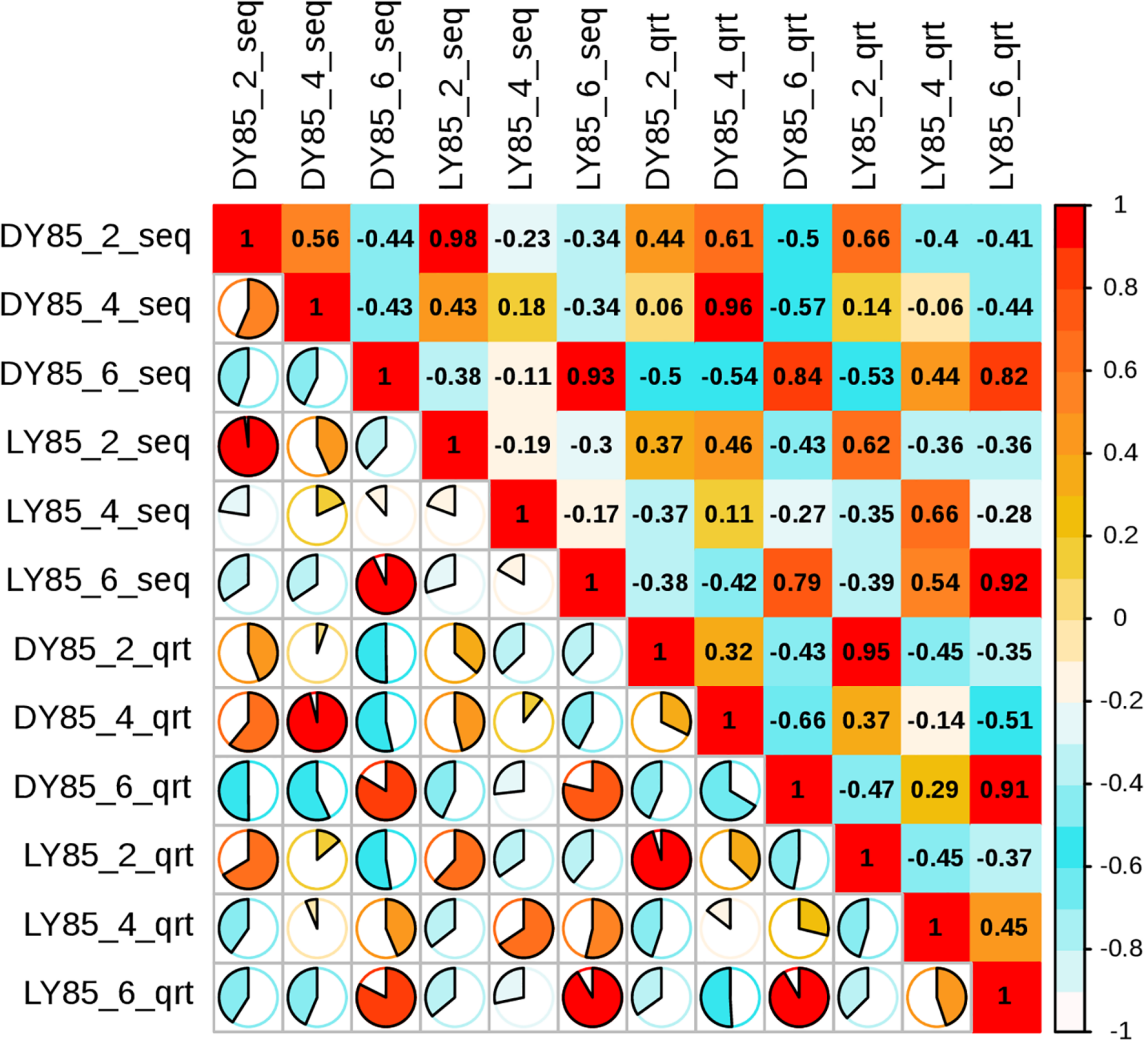
Pearson correlation analysis between relative transcript levels by qRT-PCR and transcriptome expression levels by FPKM value.

To validate the accuracy of the sequencing data, the expression levels of 14 genes were analyzed by qRT-PCR analysis, including 9 up-regulated and 5 down-regulated genes. 7 of the 9 up-regulated genes and 3 of the 5 down-regulated genes were shown consistent expression trends (**Figure S12**). Pearson correlation coefficient shows that there is correlation between transcriptome data and QRT-PCR validation results (**Figure 10**). These results reinforce the reliability of the sequencing data.

## Discussion

Light is one of the important environmental factors in the life cycle of higher plants. Photothermal receptor phytochromes integrate light and temperature signals (Jung et al., 2016; Legris et al., 2016), thereby regulating plant growth and development to adapt to the environment. For instance, light‐activated phytochrome to regulate the degradation of its direct targets PIFs, resulting in increasing of GA level and decreasing of ABA level (Oh et al., 2006; Oh et al., 2007), promoting germination. Light‐activated phytochrome regulate the degradation of PIFs and the destabilize of COP1/SPA complexes (Al-Sady et al., 2006; Shen et al., 2007; Huang, 2014; Hoecker, 2017)), resulting in the accumulation of HY5 (Osterlund et al., 2000), which modulates photomorphogenesis. Modulate of light, PHYs and PIFs plays important roles in both germination and post-germination development of *Arabidopsis* seed. However, the molecular mechanisms of germination do differ among seed categories. Photophilic seeds germinate well under light exposure, while photophobic seeds favor germinate in the dark. In this study, we systematically analyzed the molecular mechanisms of light regulated germination and post-germination development in shallow photodormant tobacco seeds.

### Expression pattern of genes related to light signal during seed germination and post- germination development

Germination of photophilic seeds promoted by light that had been reported in many species, such as *Arabidopsis* (Borthwick et al., 1952; Shinomura et al., 1996; Hennig et al., 2001), *Lactuca sativa* Linn (*lettuce*) (Toyomasu et al., 1998; Sawada et al., 2008), *Nicotiana tabacum* L (tobacco) (Wang et al., 2021; Dong et al., 2022), and so on. In this study, we noticed shallow photodormant seeds have higher germination percentage and faster speed under light. It is well known that GA and ABA antagonistically regulate seed germination (Bentsink and Koornneef, 2008). The interaction among light, ABA and GA has been elaborately illuminated during seed germination in *Arabidopsis*, the biosynthesis of GA is stimulated by light, and in turn high levels of GA suppresses the synthesis of ABA (Vanstraelen and Benková, 2012; de Wit et al., 2016). Unexpectedly, no genes (proteins) related to this pathway were found to be differentially expressed by combined transcriptome and proteome analysis in this study.

In *Arabidopsis*, light promotes the expression of *GA3ox1* (GA biosynthesis) and *CYP707A2* (ABA catabolism) and inhibits the transcription of the *GA2ox2* (GA catabolism) and *NCED6, 9* (ABA biosynthesis), resulting in a high GA/ABA ratio and consequently promoting seed germination (Kim et al., 2008; Gabriele et al., 2009; Née et al., 2017). In this study, the expression of *NtGA3ox2* and *NtCYP707A1* is promoted, and *NtNCED6* is suppressed by light (Fig S13). In addition, the expressions of *GIBBERELLIC ACID-IN-SENSITIVE* (*GAI*, a negative regulator of GA signal) (Oh et al., 2007) and *ABSCISIC ACID-INSENSITIVE 3, 5* (*ABI3*, *5*, positive regulators of ABA signal) (Oh et al., 2009; Kim et al., 2016) are inhibited by light to promote seed germination in *Arabidopsis*. In this study, the expression of *NtGAI* and *NtABI3*, *5* is suppressed by light (**Figure S13**). Similar results were noticed in other photophilic-seeded species, such as lettuce (Toyomasu et al., 1998; Sawada et al., 2008), and tobacco (Wang et al., 2021; Dong et al., 2022).

Therefore, we believed that the light signaling pathway also plays a key regulatory role in the germination of shwallow photodormant seeds. In our previous study, a large number of DEGs in this signaling pathway were detected during germination of shwallow photodormant seeds in tobacco by signal transcriptomic sequencing (Dong et al., 2022). Why these DEGs (DEPs) could not be detected in this study? Possibly because less number of DEPs was detected by proteomics sequencing and some DEGs or (DEPs) were filtered by the combined analysis of transcriptome and proteome.

### The role of light on cell wall hydrolysis during seed germination

It has been hypothesized that light regulates germination of tobacco seed in two aspects (Leubner-Metzger, 2003). On the one hand, light activates phytochromes to release photodormancy and promote germination. On the other hand, light promotes endosperm rupture in non-photodormant seeds. Several specific target enzymes, such as EXP (EXPANSIN), XTH (XYLOGLUCANENDO-TRANSGLYCOSYLASE/HYDROLASE), and βGLUI (CLASS β-1, 3-GLUCANASE), have been proposed to promote germination by inducing endosperm weakening in *Arabidopsis* and tobacco (Steinbrecher and Leubner-Metzger, 2016). The expressions of genes required for cell wall loosening, such as *EXP* and *XTH*, are repressed to inhibit seed germination under dark in *Arabidopsis* (Oh et al., 2009). The expression of *NtXTH* was upregulated by light to promote the germination of fresh seeds in tobacco (Wang et al., 2021). The above studies suggest that light can promote the weakening of endosperm during seed germination by inducing the expression of genes involved in regulating cell wall hydrolysis.

In this study, 6 MANs were up-regulated during radicle protrusion, whose functions were predicted to regulate mannosidase activity and hydrolase activity of hydrolyzing O-glycosyl compounds and glycosyl bonds. βGLUI is induced prior to the rupture of the micropylar endosperm, which is required for germination of tobacco seeds (Leubner-Metzger and Meins, 2000; Petruzzelli et al.,1999), and light is hypothesized to be involved in the induction of βGLUI (Wu et al., 2001). However, expression levels of MANs were not significantly improved by light. So, we speculated that cell wall hydrolysis is required for seed germination, and there may be two types of hydrolases, one is regulated by light, and the other not be affected by light. This also helps explain that nearly half of the shallow photodormant seeds can germinate in dark, while light can improve the germination percentage and accelerate germination speed.

### The role of light in chloroplast development during seed germination and post germination development

Seed germination begins with imbibition and completes at radicle protrusion, and then enters the stage of seedling development, including hypocotyl elongation, cotyledon expansion and de-yellowing, etc. In soil, seeds germinate in dark and develop following a dark-adapted biology program named skotomorphogenesis, which is characterized by long hypocotyls and etiolated cotyledons that contain etioplasts. When the seedlings emerge from the soil, the developmental program changes into photomorphogenesis, hypocotyl elongation is inhibited, and the cotyledons turn green and develop functional chloroplasts (Pogson et al., 2015). In the present study, similar phenotypes were noticed when the seeds were incubated in light and dark.

From quiescent seeds to imbibed seeds (DAI2), the shoot apical meristem and epidermis are completely devoid of chlorophyll, regardless of their light environment (Tejos et al., 2010; Yadav et al., 2019). In this study, the phenotypic difference was not significant on DAI2, and photosynthesis-related genes were not differentially expressed by light. When the radicle protrudes, photosynthesis-related genes (proteins) were up-regulated or down-regulated by light, followed by a green hypocotyl phenotype. In direct photomorphogenesis, cell-specificity of chloroplast biogenesis of mostly cotyledons starts after the emergence of radicle from the seed coat (Yadav, 2019). In this study, multiple regulators involved in photosynthesis, such as GAPA1, 2, GAPAB, GAPC1, 2, PSBO2 and CP12-2, were noticed to be regulated by light at radicle protrusion, but not at cotyledon expansion stage. Notably, 18 of 23 hut genes (proteins) are located in the chloroplast at radicle protrusion stage.

Therefore, we speculated that chloroplast development genes were highly expressed after radicle protrusion, which may lay the foundation for photosynthesis in subsequent seedling development.

Light is an important signal factor that involved in regulating of chloroplast biogenesis during post-germination development. It requires the coordinated expression of chloroplast biogenesis genes encoded in nuclear and plastid genomes (Liebers et al., 2022). PHYB are activated by light to promote the degradation of PIF3, which represses the nuclear-encoded components of the plastid transcriptional machinery required for transcription of the plastid-encoded photosynthesis genes (Dubreuil et al., 2017). The light-PHYs-PIFs module not only plays an important role in seed germination and post-germination development, but also participates in the regulation of chloroplast development. It has been widely concerned the relationship between seedling morphogenesis and chloroplast biogenesis. For instance, SCO1 is critical for plastid development during embryogenesis, and its mutants *sco1-2* and *sco1-3* display a seedling lethal phenotype. Seedlings of *sco1* mutants typically die shortly after germination due to improper chloroplast development (Ruppel and Hangarter, 2007). However, little is known about whether abnormalities in precursor, corpus luteum or chloroplast development affect seed germination. In addition, it remains unknown whether chloroplast or its precursors are involved in the regulation of seed germination.

## Conclusion

Light is an important signaling factor, which is widely involved in the regulation of germination and early seedling development in shallow photodormant tobacco seeds, including increased germination rate, inhibition of hypocotyl elongation, promotion of cotyledon unfolding, and so on. 9, 161, 342 differentially expressed genes (DEGs) and 128, 185, 81 differentially expressed proteins (DEPs) were identified at the stage of seed imbibition, radicle protrusion and cotyledon expansion, respectively. PCA results of these DEGs (DEPs) showed that samples of imbibed seeds and germinated seeds were gathered for a short distance, while the samples of post-germination seedling were completely separated from them. 0, 19 and 1 co-up-regulated, while 1, 30 and 64 co-down-regulated DEGs (DEPs) were identified at the stage of seed imbibition, radicle protrusion and cotyledon expansion, respectively, and 0, 16 and 21 pathways were enriched by using these co-up or co-down-regulated DEGs (DEPs). Oleosin 18.5 kDa (OLEO1), Glyceraldehyde-3-phosphate dehydrogenase (GAPA1), Oxygen-evolving enhancer protein 1-1 and anchloroplastic (PSBO1) were the hub genes (proteins) of these co-up- and co-down-regulated DEGs (DEPs) at radicle protrusion stage, 18 of these 23 PPI (genes) proteins were localized in the chloroplast. OLEO1 was the hub genes (proteins) of these co-up- and co-down-regulated DEGs (DEPs) at cotyledon expansion stage, and the cellular components of the more gene (protein) localization were extracellular region, nucleus and endoplasmic reticulum. The above results systematically analyzed the regulatory mechanism of light during germination and post-germination development in shallow photodormant tobacco seeds.

## Data availability statement 

The data presented in the study are deposited in the NCBI repository, accession number PRJNA914116, https://www.ncbi.nlm.nih.gov/sra/PRJNA914116.

## Author contributions

ZL supervised the research. QL, MZ, SD and PY prepared the samples and performed the experiments. ZL, QL, and JZ analyzed the data. ZL, EL and QL wrote and revised the manuscript. All authors contributed to the article and approved the submitted version.

## Supplementary material

Supplementary Figure 1 Perimeter dynamic curve of seed germinated under light and dark.

Supplementary Figure 2 Area dynamic of seed germinated under light and dark.

Supplementary Figure 3 Number of DEGs (DEPs) of seed germinated under light and dark.

Supplementary Figure 4 Enriched GO terms of light-regulated pathways at cotyledon expansion stage. The size of the circle represents the number of genes enriched in the pathway, and the color of the cluster represents the confidence interval, and *p*-value was calculated from hypergeometric test.

Supplementary Figure 5 Multiple comparisons of light-regulated DEGs (DEPs) during seed germination and post-germination development. The histogram on the left represents the number of co-up and co-down regulated DEGs (DEPs) within each comparison. The histogram on the top represents the number of co-up or -down regulated DEGs (DEPs) from multiple comparisons. Multiple comparisons are displayed by lines and linked dots.

Supplementary Figure 6 Enriched GO terms of DEGs (DEPs) from sko-germinated seeds/sko-imbibed seeds. The size of the circle represents the number of genes enriched in the pathway, and the color of the cluster represents the confidence interval, and *p*-value was calculated from hypergeometric test.

Supplementary Figure 7 Enriched GO terms of DEGs (DEPs) from sko-developed seedling/sko-germinated seeds. The size of the circle represents the number of genes enriched in the pathway, and the color of the cluster represents the confidence interval, and *p*-value was calculated from hypergeometric test.

Supplementary Figure 8 Enriched GO terms of DEGs (DEPs) from photo-germinated seeds/photo-imbibed seeds. The size of the circle represents the number of genes enriched in the pathway, and the color of the cluster represents the confidence interval, and *p*-value was calculated from hypergeometric test.

Supplementary Figure 9 Enriched GO terms of DEGs (DEPs) from photo-developed seedling/photo-germinated seeds. The size of the circle represents the number of genes enriched in the pathway, and the color of the cluster represents the confidence interval, and *p*-value was calculated from hypergeometric test.

Supplementary Figure 10 PPI network of light-regulated seeds and seedlings. **(A)** sko-germinated seeds/sko-imbibed seeds **(B)** photo-germinated seeds/photo-imbibed seeds **(C)** sko-developed seedling/sko-germinated seeds. **(D)** photo-developed seedling/photo-germinated seeds. The protein-protein interaction network for DEGs (DEPs) was analyzed using the GeneMANIA data base during seed germination and post-germination development. At least 20 most frequently changed neighboring genes are shown. Each network node represents a gene. The node color represents the possible functions of these respective genes.

Supplementary Figure 11 Cellular components from different development stages seeds. **(A)** sko-germinated seeds/sko-imbibed seeds **(B)** photo-germinated seeds/photo-imbibed seeds **(C)** sko-developed seedling/sko-germinated seeds. **(D)** photo-developed seedling/photo-germinated seeds. Petal size shows the number of cellular components enriched, and the number of signaling pathways enriched in cellular components is also listed.

Supplementary Figure 12 Expression levels of genes involved in photosynthesis during germination and post-germination development.

Supplementary Figure 13 Expression levels of genes involved in ABA or GA metabolism or signal during germination.
