# Supplementary figures and images for: Systematic analysis of photo/sko-regulated germination and post-germination development of shallow photodormant seeds in Nicotiana tabacum L
Source: Front Plant Sci. 2023 Jan 4;13:1042981. doi: 10.3389/fpls.2022.1042981 (PMC9875545; doi:10.3389/fpls.2022.1042981)

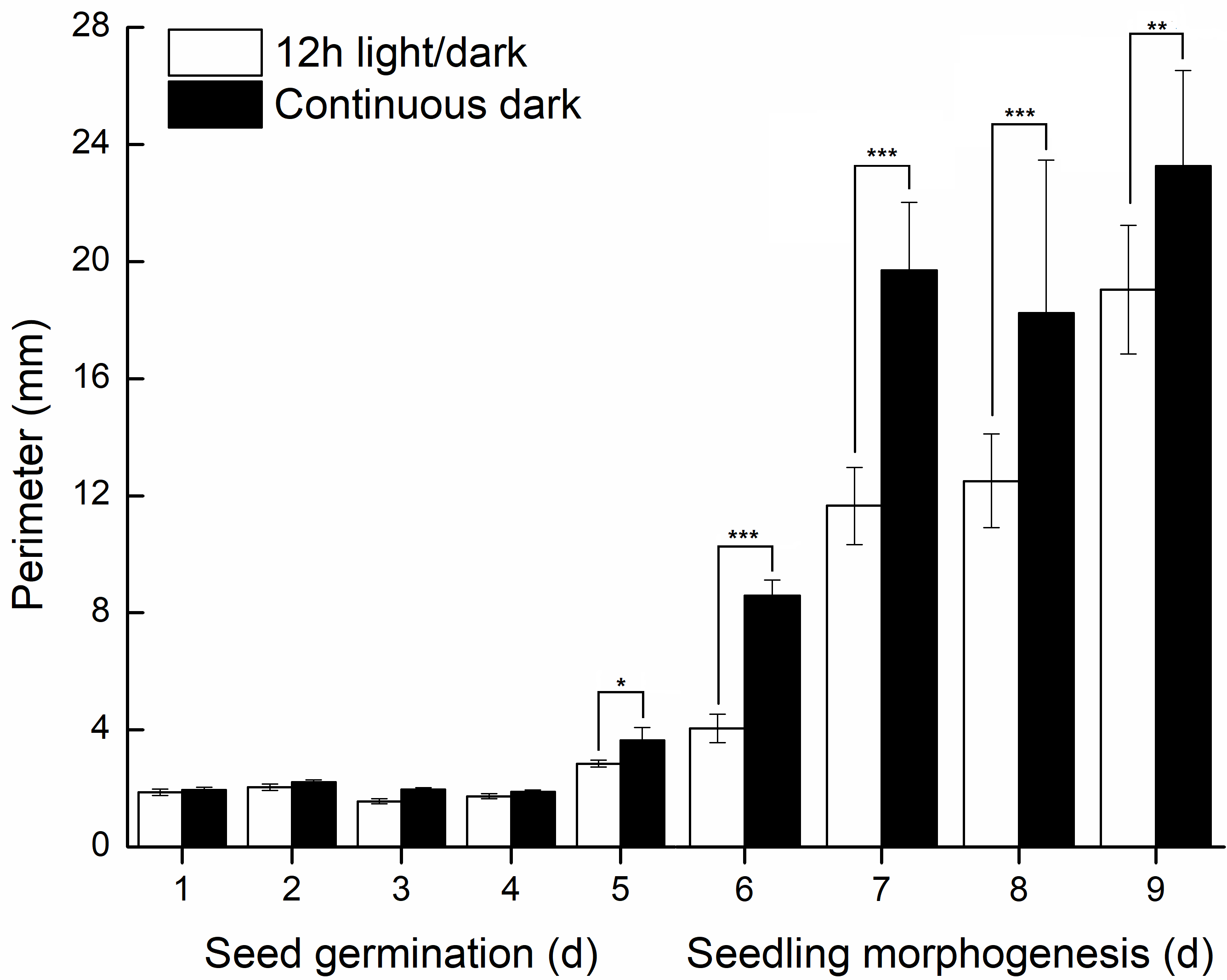

Supplement: Supplementary Figure 1 — Perimeter dynamic curve of seed germinated under light and dark. [file DataSheet_1.zip › Image 1.TIF]

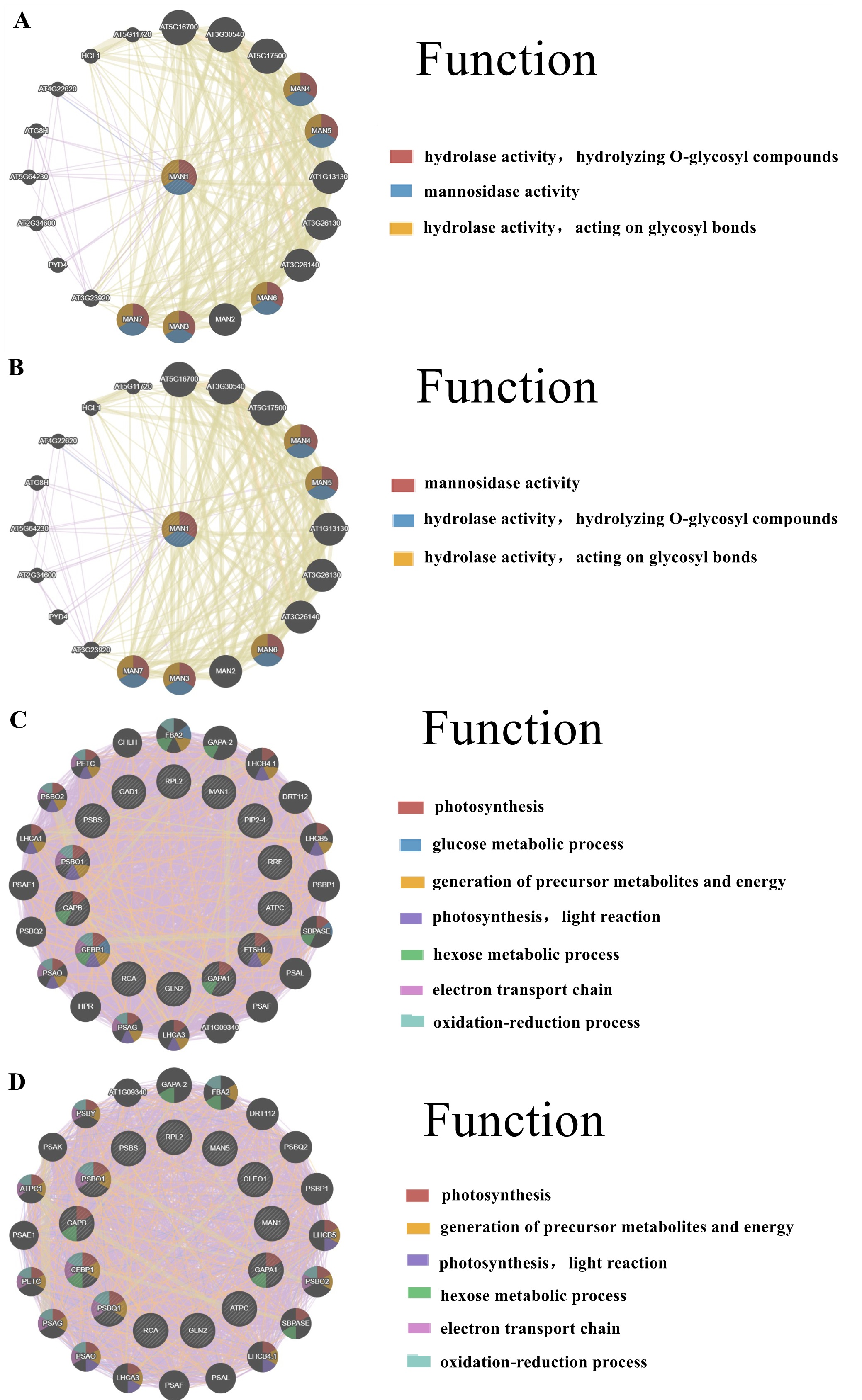

Supplement: Supplementary Figure 1 — Perimeter dynamic curve of seed germinated under light and dark. [file DataSheet_1.zip › Image 10.JPEG]

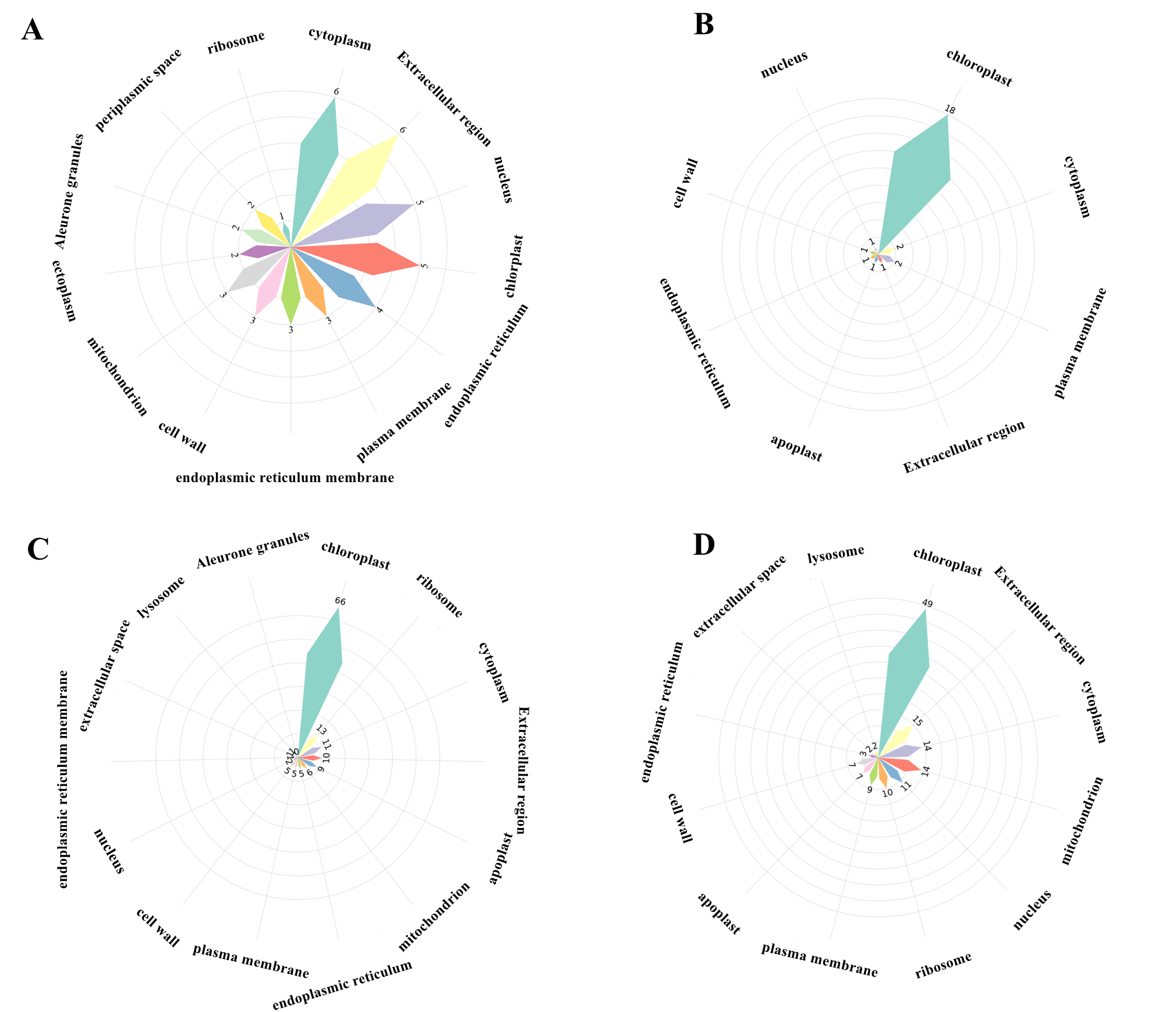

Supplement: Supplementary Figure 1 — Perimeter dynamic curve of seed germinated under light and dark. [file DataSheet_1.zip › Image 11.TIFF]

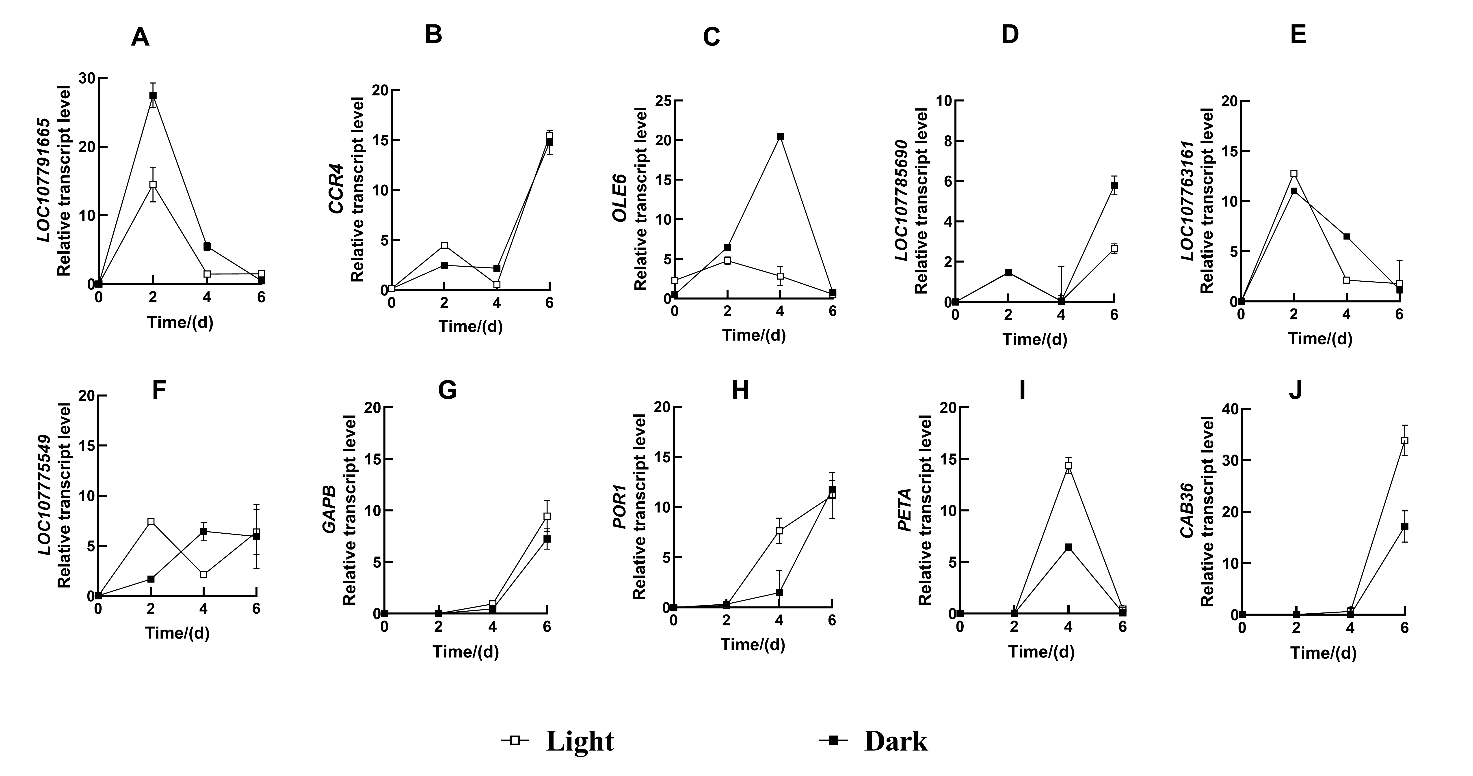

Supplement: Supplementary Figure 1 — Perimeter dynamic curve of seed germinated under light and dark. [file DataSheet_1.zip › Image 12.TIFF]

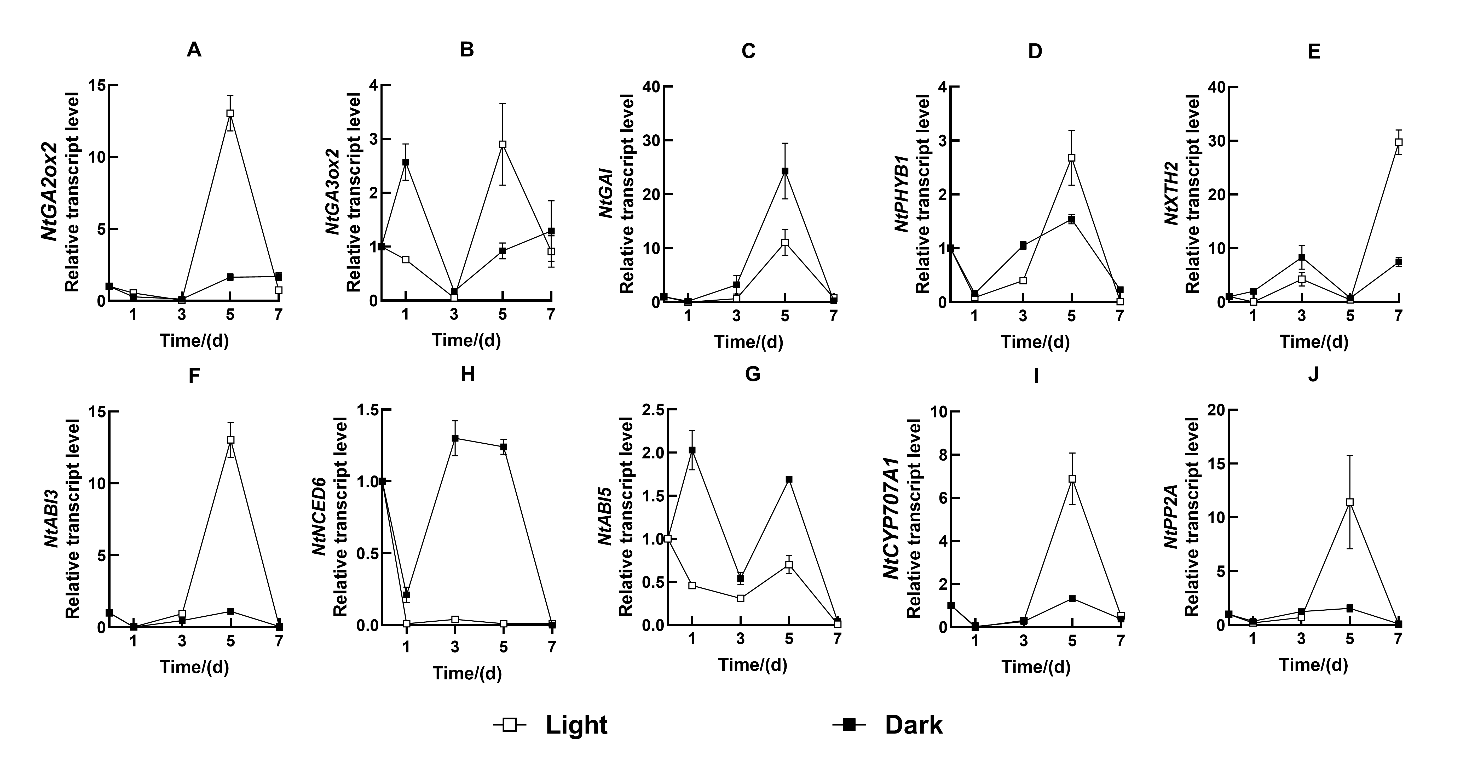

Supplement: Supplementary Figure 1 — Perimeter dynamic curve of seed germinated under light and dark. [file DataSheet_1.zip › Image 13.TIFF]

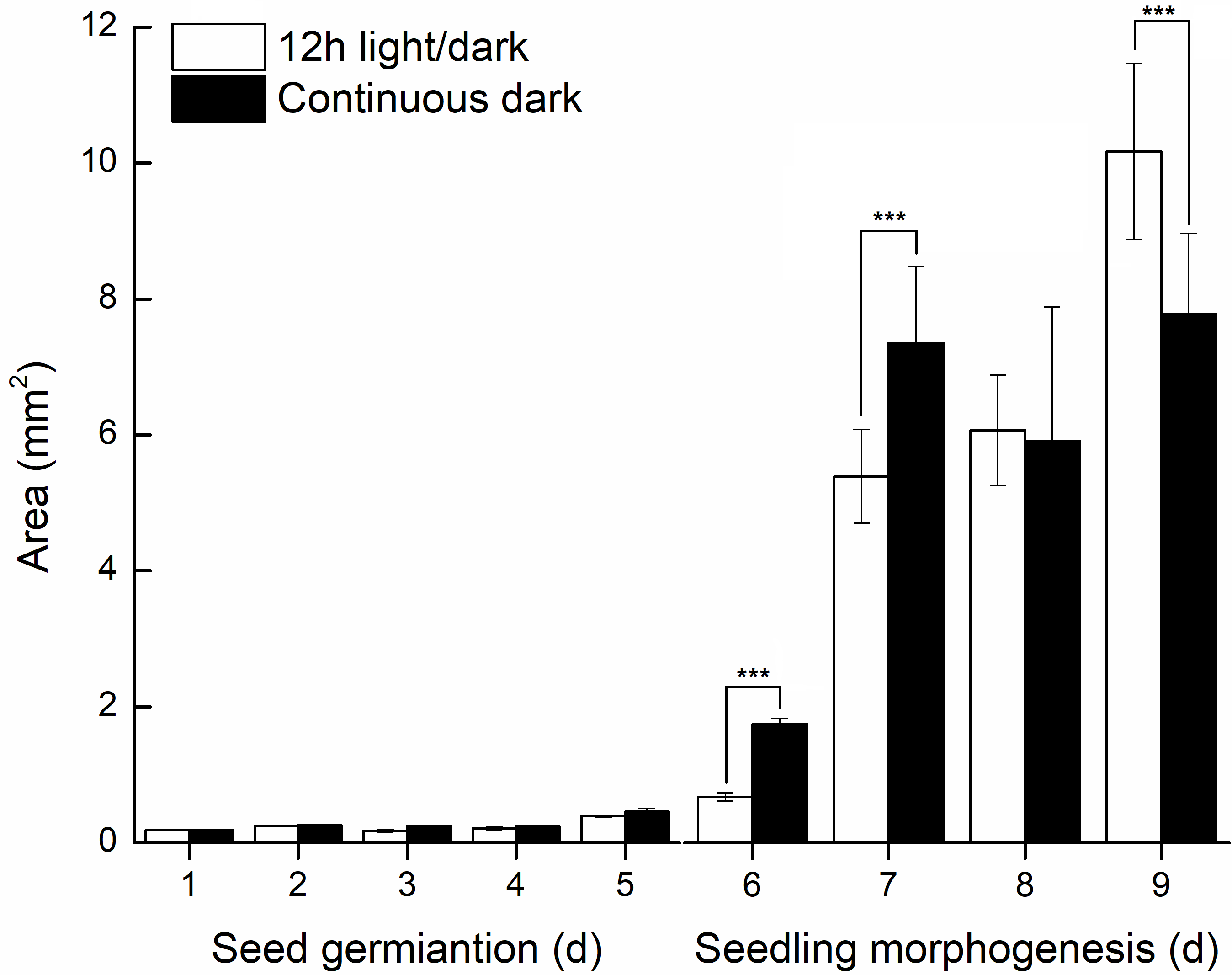

Supplement: Supplementary Figure 1 — Perimeter dynamic curve of seed germinated under light and dark. [file DataSheet_1.zip › Image 2.TIF]

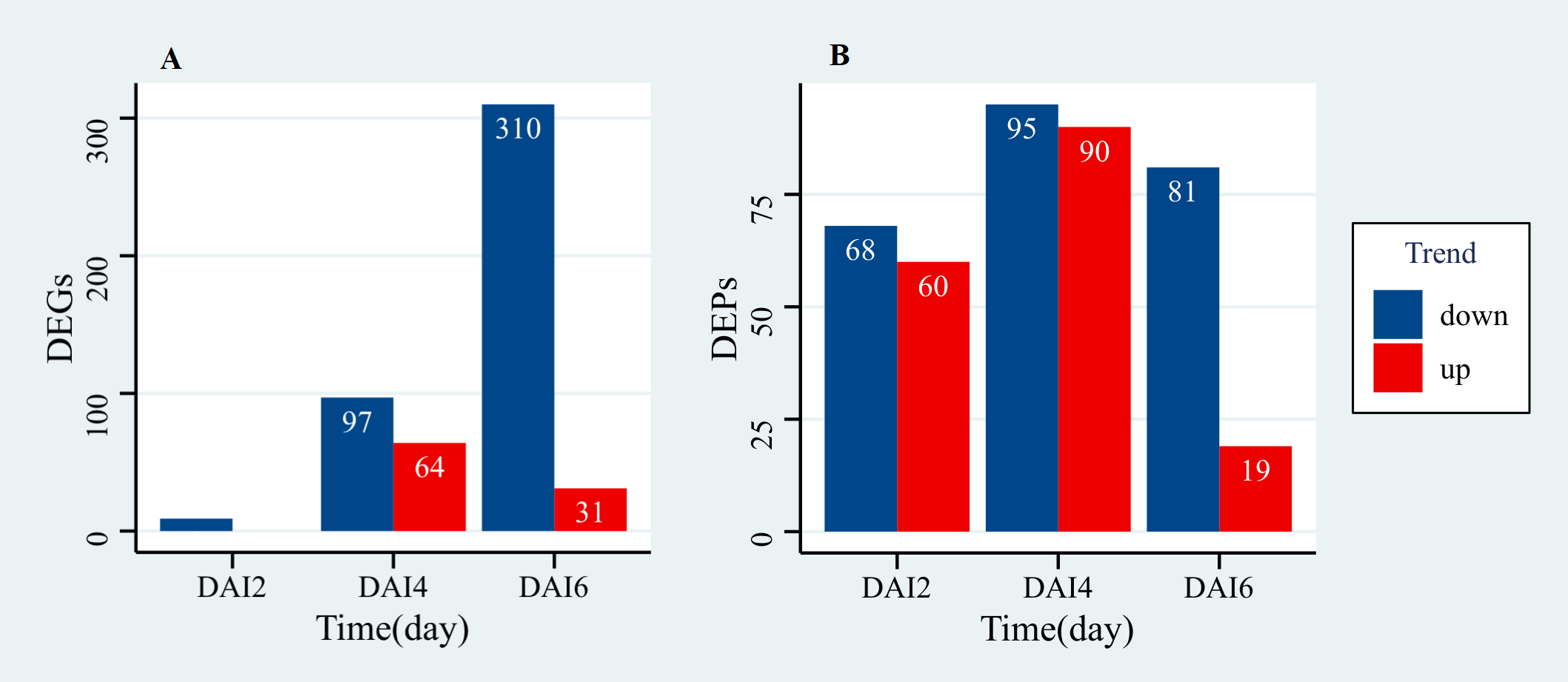

Supplement: Supplementary Figure 1 — Perimeter dynamic curve of seed germinated under light and dark. [file DataSheet_1.zip › Image 3.TIFF]

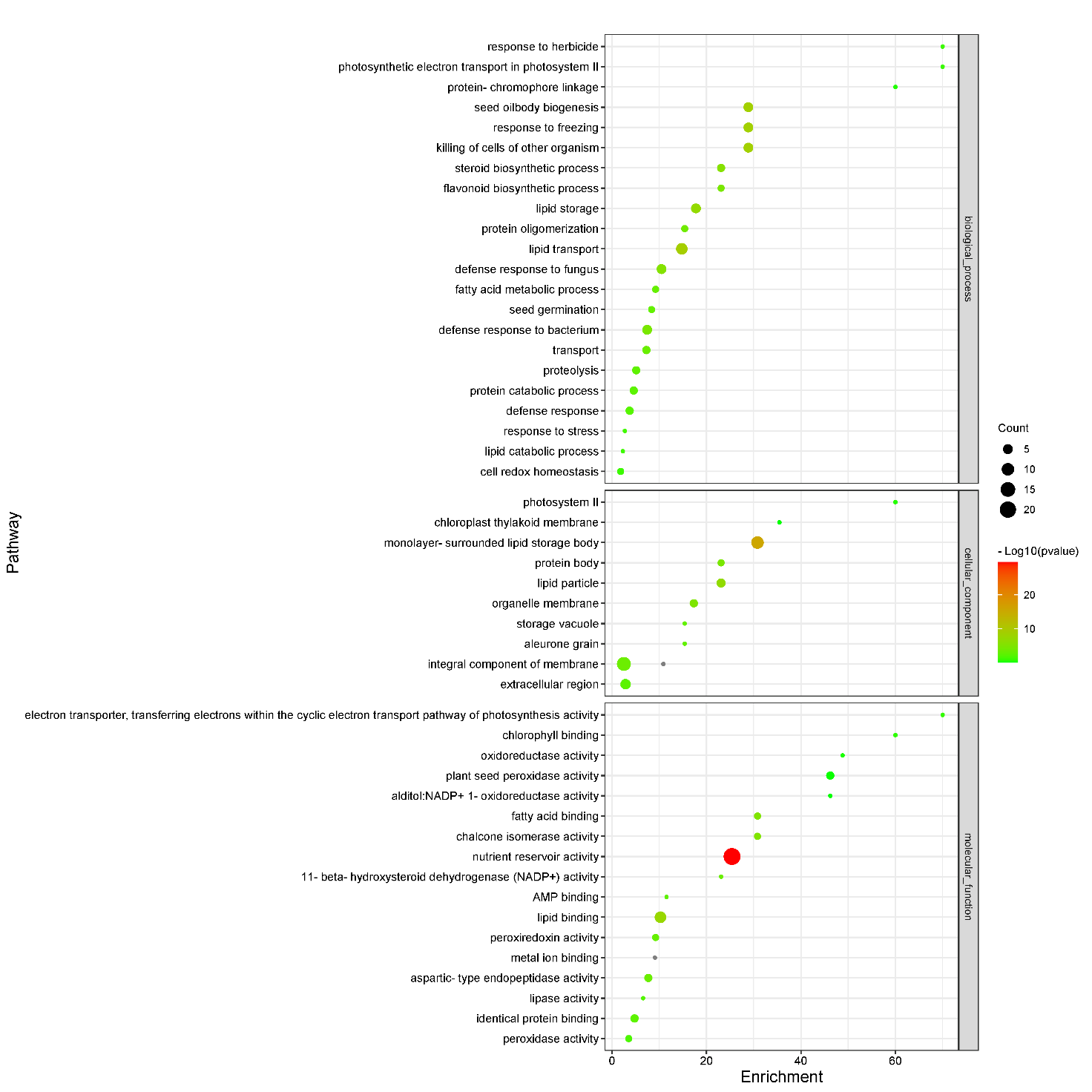

Supplement: Supplementary Figure 1 — Perimeter dynamic curve of seed germinated under light and dark. [file DataSheet_1.zip › Image 4.TIFF]

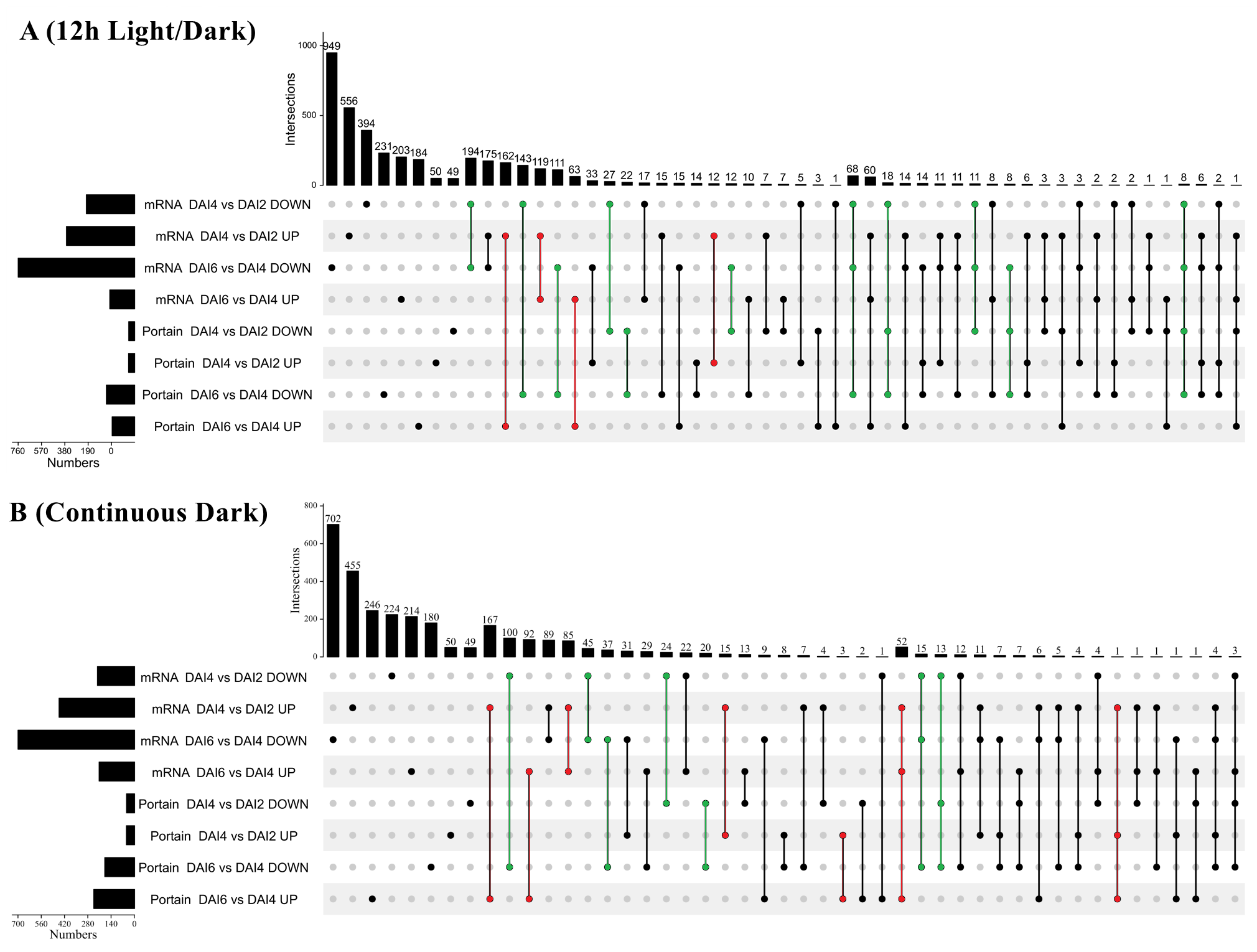

Supplement: Supplementary Figure 1 — Perimeter dynamic curve of seed germinated under light and dark. [file DataSheet_1.zip › Image 5.TIF]

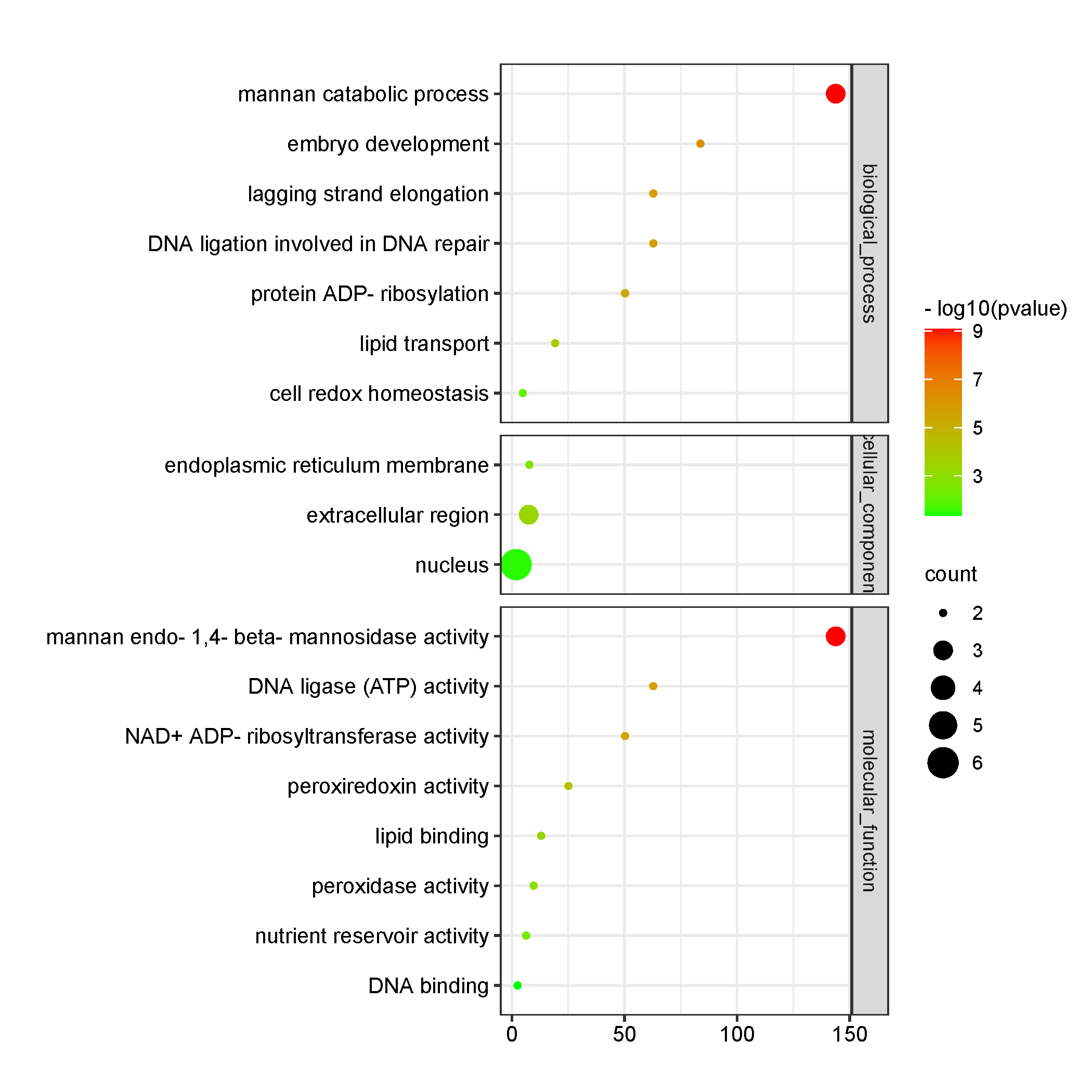

Supplement: Supplementary Figure 1 — Perimeter dynamic curve of seed germinated under light and dark. [file DataSheet_1.zip › Image 6.TIFF]

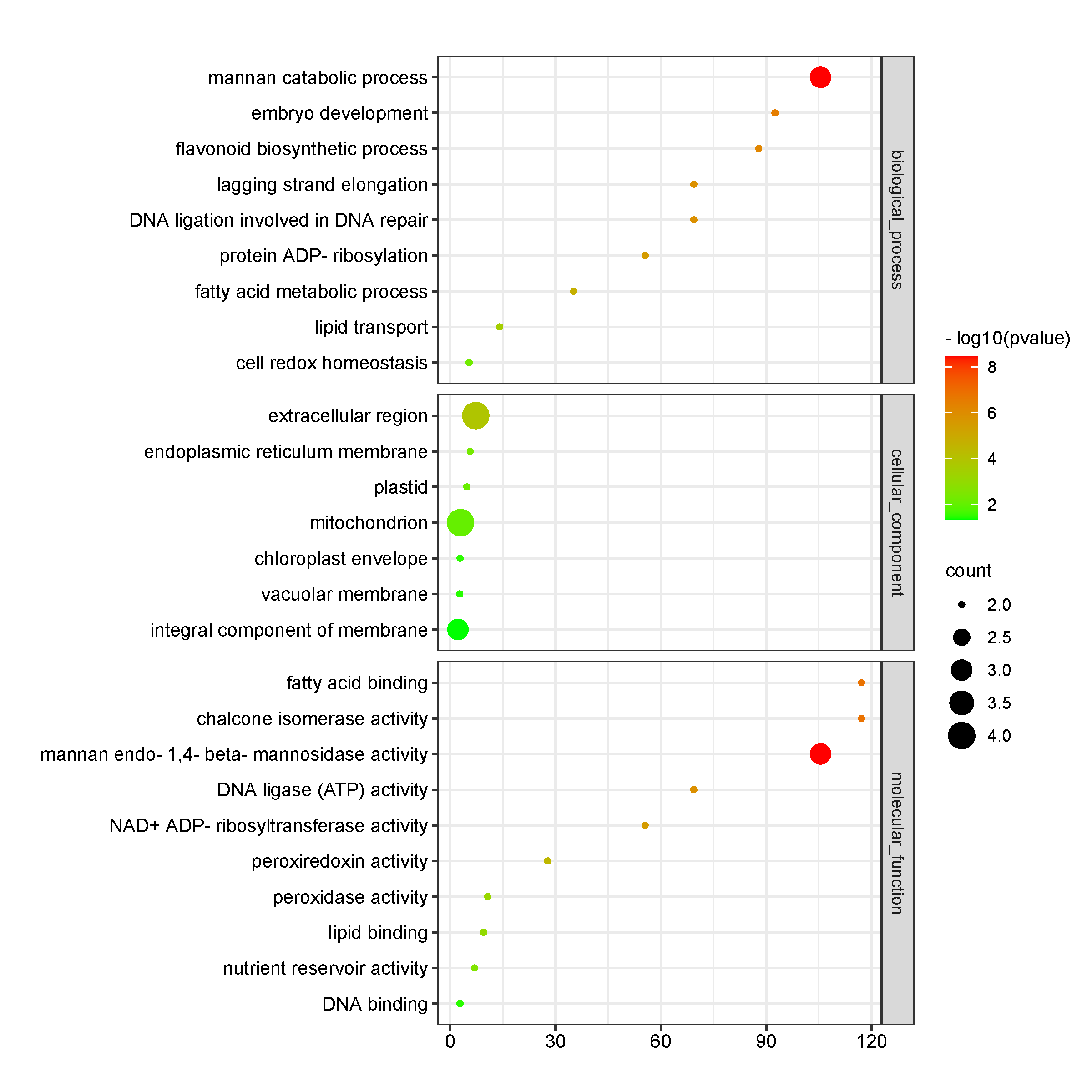

Supplement: Supplementary Figure 1 — Perimeter dynamic curve of seed germinated under light and dark. [file DataSheet_1.zip › Image 7.TIFF]

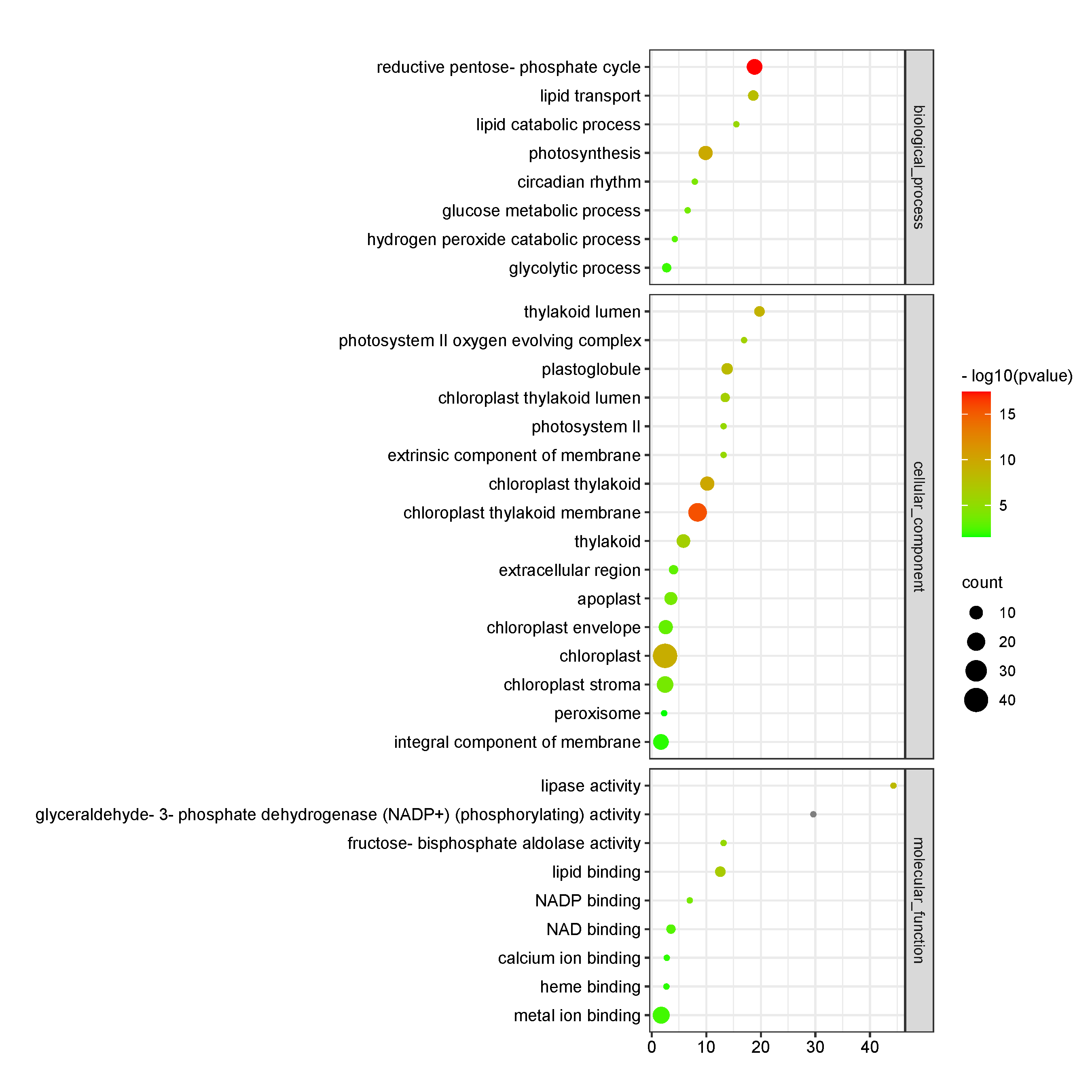

Supplement: Supplementary Figure 1 — Perimeter dynamic curve of seed germinated under light and dark. [file DataSheet_1.zip › Image 8.TIFF]

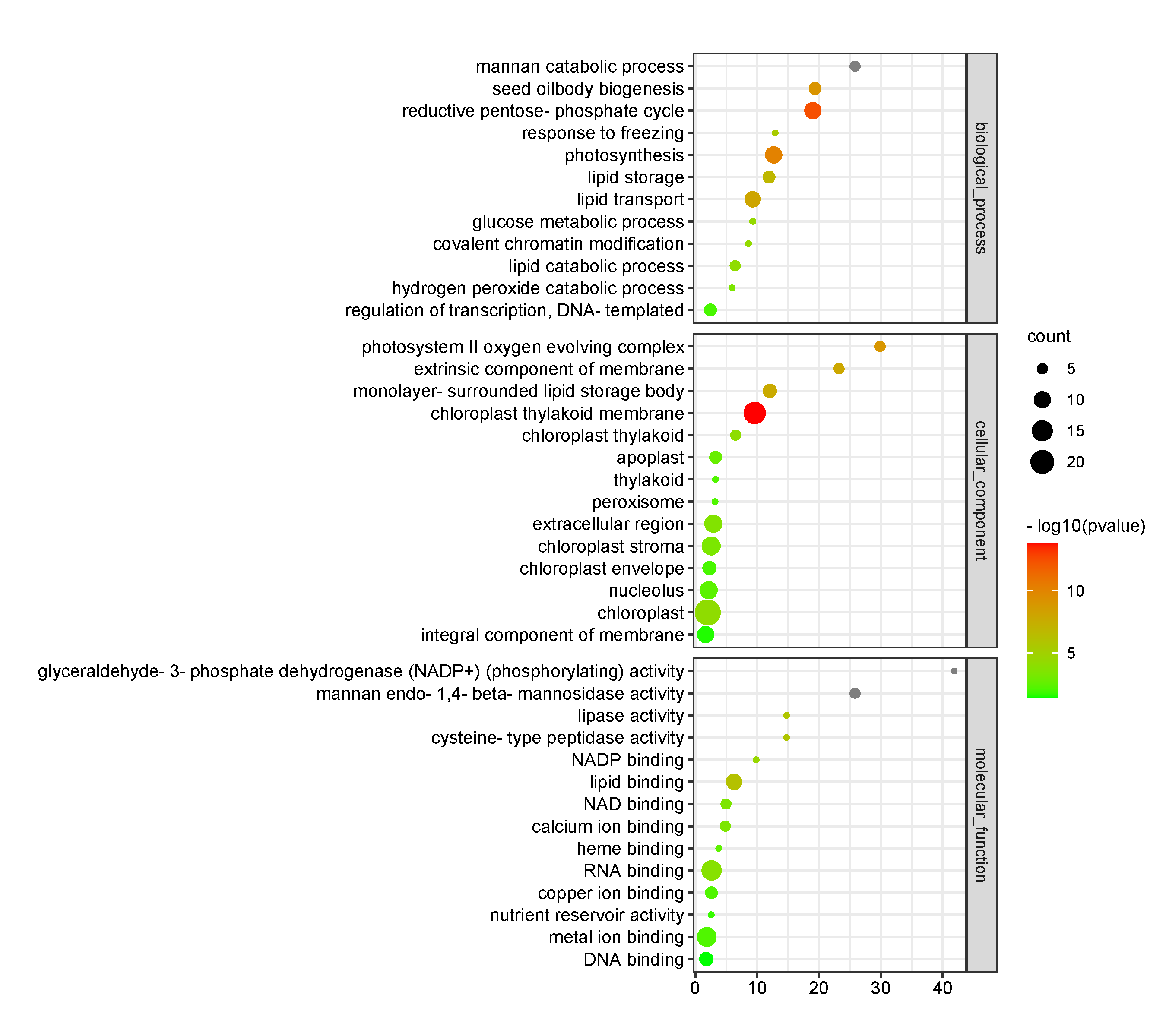

Supplement: Supplementary Figure 1 — Perimeter dynamic curve of seed germinated under light and dark. [file DataSheet_1.zip › Image 9.TIFF]
