# Supplementary material for: Systematic analysis of photo/sko-regulated germination and post-germination development of shallow photodormant seeds in Nicotiana tabacum L
Source: Front Plant Sci. 2023 Jan 4;13:1042981. doi: 10.3389/fpls.2022.1042981 (PMC9875545; doi:10.3389/fpls.2022.1042981)
Supplement: Supplementary file 2 [file Table_1.docx]

| **TABLE S1 Real-time PCR primers used for genes expression analysis.** | | |
| --- | --- | --- |
| **Primer Name** | **Primer Sequence (Forward)** | **Primer Sequence (Reverse)** |
| *NtGA3ox2* | TGGAAAAACTAGCCGGAAGA | GCCCATTTCATATCGTCCTTAC |
| *NtGA2ox2* | TTGGAGGACCACCATTGAGT | CAAGCTGTCTTGATCCCCTTT |
| *NtGAI* | TCCACTAACAACAGATGCAACAACAAG | ACAGCTTCAGCACACGCCATT |
| *NtNCED6* | AGTTTCGGGTTGGTGGATGCTAC | CTGTAATACGGACGCTATACGGAAGAT |
| *NtCYP707A1* | GGTGATTCTGCTGGTGTTGTCTCT | GGGATATAGCTTAATGGGCAGA |
| *NtABI3* | GAGTATCAGACCATGGAATCTGC | TTCCATCGCGGAGAATTG |
| *NtABI5* | CGCAAAAGGCGACTAACAA | ACACATCAAGGGCAACTCAA |
| *NtXTH2* | GGCTAGTCACCACATCAAGTACCTCA | CACCTGAAGACCTGTCAAGAACAAGAT |
| *NtTOC1* | TGCTTCCACCACTGCTGCTCATA | TCCTGTCTGCCGTTCATTAGTTCCT |
| *NtPHYB1* | GTGTGATACTGTGGTTGAGAGTGTGA | TTGAGGAATGTCGGTAGCAGGATAATG |
| *NtCCR4* | TTGGCAAGAATCCGGTGTGAATCC | TTGACGGTGGCATTGGCTGTG |
| *NtOLE6* | ACGGGCATAGACTACGGCGATAC | TGCTGCTCCTTCACTGTCTCAATTC |
| *LOC107785690* | GGAACTCGGAGGAAAGGATGCTTG | TTCCATCACCAACACGACCTTAACG |
| *NtCAB36* | GACACTGATAGGCTCGCTCTTGAC | AAGACACAGCCCAAAGCACCAAG |
| *LOC107763161* | GATCCCATGCCAGCCAGATGTG | CAAGCCAAGACCTAACTCCACCAG |
| *LOC107775549* | TGGACACAAACTTCACAGTCGGAAC | GCTGCTGCTACTTGATCTCTTCCTG |
| *LOC107791665* | GATGCTGTTAATGCTTGGGCTGATG | CCACAAAGTTGCCCTTCAACACAAG |
| *NtPETA* | ACCTATTCTTTCTCCTGATCCAGC | AAAGTTGACCACGTCCACGG |
| *NtPOR1* | GCCAAAGCGTACAAAGACAGCAAG | CAATGCAGCCAGGGTAAAGAGAGG |
| *NtGAPB* | TGACTTGAGGAGAGCGAGAGCAG | GGTAGCACTAAAGACACAGCCTTGG |
